# Stereoselective amine-thiourea-catalysed sulfa-Michael/nitroaldol cascade approach to 3,4,5-substituted tetrahydrothiophenes bearing a quaternary stereocenter

**DOI:** 10.3762/bjoc.12.63

**Published:** 2016-04-05

**Authors:** Sara Meninno, Chiara Volpe, Giorgio Della Sala, Amedeo Capobianco, Alessandra Lattanzi

**Affiliations:** 1 Dipartimento di Chimica e Biologia “A. Zambelli”, Via Giovanni Paolo II, 84084, Fisciano, Italy

**Keywords:** cascade reaction, tetrahydrothiophenes, aymmetric synthesis, amine thioureas, organocatalysis

## Abstract

An investigation on the stereoselective cascade sulfa-Michael/aldol reaction of nitroalkenes and commercially available 1,4-dithiane-2,5-diol to 3,4,5-substituted tetrahydrothiophenes, bearing a quaternary stereocenter, is presented. A secondary amine thiourea derived from (*R*,*R*)-1,2-diphenylethylamine was found to be the most effective catalyst when using *trans*-β-methyl-β-nitrostyrenes affording the heterocyclic products in good yields and moderate stereoselectivities.

## Introduction

The interest toward the development of stereoselective methodologies to prepare tetrahydrothiophenes bearing multiple chiral centers increased over the last years [1]. Indeed, chiral non-racemic functionalized tetrahydrothiophenes are endowed with different biological activities [2–5] and they are useful ligands in asymmetric catalysis [6]. However, few asymmetric approaches are available to obtain this class of compounds and only recently organocatalytic stereoselective cascade reactions have emerged as the most successful, and straightforward approach to access them [7–8]. Aminocatalytic [9–12] and non-covalent organocatalytic cascade sulfa-Michael/Michael [13–15] and sulfa-Michael/aldol reactions [16–20] enabled the synthesis of differently functionalized tetrahydrothiophenes bearing up to three contiguous chiral centers, including quaternary ones, with good to high control of the diastereo- and enantioselectivity.

Surprisingly, to date there has been one report on a dynamic system combining a 1,1,3,3-tetramethylguanidine (TMG)/ZnI_2_-catalyzed diastereoselective cascade sulfa-Michael/nitroaldol reaction followed by lipases catalyzed kinetic resolution using two representative *trans*-β-methyl-β-nitrostyrenes and 1,4-dithiane-2,5-diol as reagents [21]. One diastereoisomer of the racemic tetrahydrothiophenes, present at the equilibrium, was preferentially acylated by the enzyme to give the product in high ee.

Different amines such as Et_3_N, DBU, TMG catalyze the sulfa-Michael/nitroaldol process of either *trans*-β-methyl-β-nitrostyrenes [21] and *trans*-β-nitrostyrenes [22] with 1,4-dithiane-2,5-diol. Based on all above considerations and prompted by our interest in asymmetric synthesis of functionalized tetrahydrothiophenes [14], we wondered whether we could use bifunctional organocatalysts to develop a diastereo- and enantioselective cascade sulfa-Michael/nitroaldol reaction and herein we report our preliminary results.

## Results and Discussion

According to the literature data, low control of the diastereoselectivity was observed in the cascade reaction of 1,4-dithiane-2,5-diol with *trans*-β-nitrostyrenes, and in the case of (*E*)-1-aryl-2-nitropropene all four diastereoisomeric tetrahydrothiophenes were observed when using tertiary amines such Et_3_N, DBU or TMG [21–22].

**Scheme 1:**
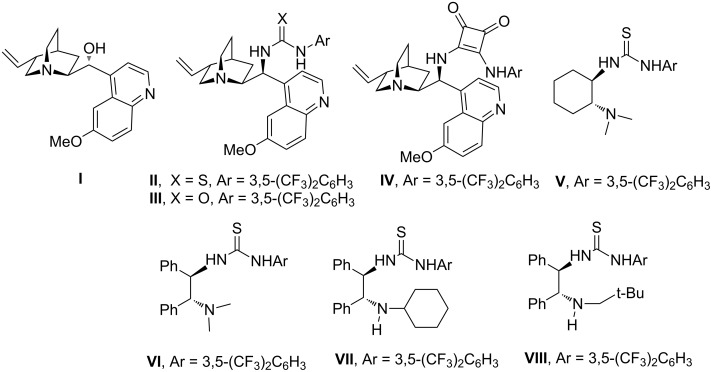
Organocatalysts screened in the cascade reaction.

At the outset, the sulfa-Michael/nitroaldol reaction was studied by reacting *trans*-β-nitrostyrene, (*E*)-1-phenyl-2-nitropropene and (*E*)-1-phenyl-2-nitrobutene in toluene at room temperature with 1,4-dithiane-2,5-diol as precursor of mercaptoacetaldehyde, using 10 mol % loading of different bifunctional organocatalysts (Scheme 1, Table 1).

**Table 1:** Asymmetric sulfa-Michael/nitroaldol reaction of nitroalkenes **1**–**3** with 1,4-dithiane-2,5-diol (**4**) catalyzed by catalysts **I**–**VIII**.

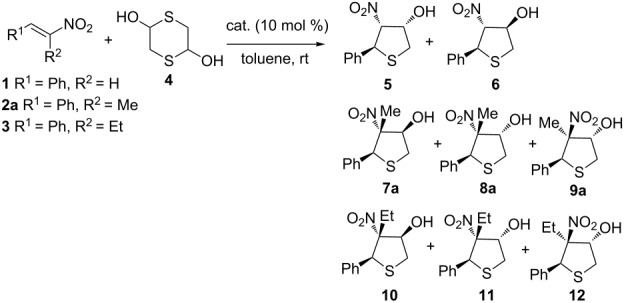						

entry	cat.	**1**–**3**	time (h)	yield [%]^a^	dr^b^	ee [%]^c^

1	**I**	**1**	5.5	75	58/42 (**5**/**6**)	9 (**5**)/15 (**6**)
2	**II**	**1**	2.5	79	60/40 (**5**/**6**)	rac (**5**)/24 (**6**)
3	**III**	**1**	1.5	53	44/56 (**5**/**6**)	rac (**5**)/12 (**6**)
4	**IV**	**1**	25	52	50/50 (**5**/**6**)	−14 (**5**)/20 (**6**)
5	**V**	**1**	2	89	38/62 (**5**/**6**)	−3 (**5**)/−10 (**6**)
6	**VII**	**1**	2	73	72/28 (**5**/**6**)	−12 (**5**)/−24 (**6**)
7	**I**	**2a**	21	77	66/28/6 (**7a**/**8a**/**9a**)	19 (**7a**)
8	**II**	**2a**	17	77	66/27/7 (**7a**/**8a**/**9a**)	13 (**7a**)
9	**III**	**2a**	17	69	68/24/8 (**7a**/**8a**/**9a**)	4 (**7a**)
10	**IV**	**2a**	27	59	70/23/7 (**7a**/**8a**/**9a**)	2 (**7a**)
11	**V**	**2a**	18	72	68/24/8 (**7a**/**8a**/**9a**)	−19 (**7a**)
12	**VI**	**2a**	44	16	69/25/6 (**7a**/**8a**/**9a**)	1 (**7a**)
13	**VII**	**2a**	28	90	58/30/12 (**7a**/**8a**/**9a**)	−44 (**7a**)
14	**VIII**	**2a**	40	70	65/29/6 (**7a**/**8a**/**9a**)	19 (**7a**)
15	**VII**	**3**	52	80	50/27/23 (**11**/**10**/**12**)	42 (**11**)

^a^Isolated yield of all diastereoisomers after silica gel chromatography. ^b^Determined by ^1^H NMR analysis of the crude mixture. ^c^Determined by chiral HPLC analysis. Negative values indicate the prevalent formation of the opposite enantiomer.

**Scheme 2:**
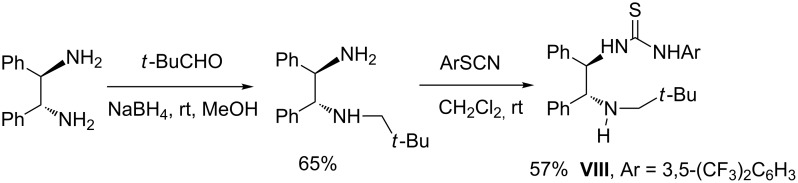
Synthesis of catalyst **VIII**.

In the case of *trans*-β-nitrostyrene (**1**), a mixture of diastereoisomers **5** and **6** were rapidly formed, irrespective of the catalyst used, with a poor level of diastereo- and enantioselectivity (Table 1, entries 1–5). Thiourea catalyst **VII** afforded the best result, leading to products **5**/**6** in a ratio of 72/28 although with low ee values (Table 1, entry 6). Reacting trisubstituted olefin **2a** with compound **4**, led to three isomers with modest diastereocontrol when using catalysts (**I–VII**). Among the catalysts tested, compound **VII** proved to be the most active and enantioselective, giving the major diastereoisomer **7a** with 44% ee (Table 1, entry 13). Taking into account that amine thiourea **VII**, bearing a sterically hindered secondary amine moiety, was significantly more effective than tertiary amine-based thioureas [14,23], catalyst **VIII**, bearing a sterically demanding neopentyl group, was synthesized in order to check its impact on the stereoselectivity (Scheme 2).

Catalyst **VIII** was easily obtained in a two-step procedure in fair overall yield. Compound **VIII** proved to be less active, affording a comparable level of diastereoselectivity than compound **VII**, but a lower ee value for major diastereoisomer **7a** was measured (Table 1, entry 14). Moreover, the opposite enantiomer of product **7a** was preferentially obtained, thus suggesting that the nature of the alkyl group on the secondary amine moiety greatly affects the stereochemical outcome of the process. The relative configuration of diastereoisomers **7a**/**8a/9a** was established by NOESY and NOE analysis on diastereoisomerically pure **7a** and on a diastereoisomeric mixture of compounds **8a** and **9a** (see the Supporting Information File 1) [24].

Finally, catalyst **VII** was checked in the reaction of alkene **3** under the same conditions (Table 1, entry 15). After a longer reaction time, diastereoisomers **10**–**12** were isolated with poor diastereomeric ratio and major isomer **11** was recoverd with 42% ee [25].

The diastereoselective ratios, determined for tetrahydrothiophenes deriving from alkenes **2a** and **3**, are in line with their computed thermodynamic stability in toluene (see the Supporting Information File 1). Indeed, products **8a** and **9a** were found to be 0.7 kcal/mol less stable compared to compound **7a**. In the case of products **10–12**, the predicted relative free energies for **10**, **11** and **12** fall within a range of 0.1 kcal/mol.

Pleasingly, a solvent screening for the cascade reaction carried out on compound **2a** with catalyst **VII** enabled to improve the diastereoselectivity as only **7a**/**8a** were detected in 75:25 ratio and the enantiocontrol increased to 50% ee for diastereoisomer **7a** when using chlorobenzene as the solvent at room temperature (Table 2, entry 5).

**Table 2:** Solvent screening in the asymmetric Michael/nitroaldol reaction of **2a** with 1,4-dithiane-2,5-diol (**4**) catalysed by amine thiourea **VII**.

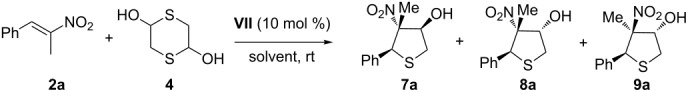					

entry	solvent	time (h)	yield [%]^a^	dr^b^	ee **7a** [%]^c^

1	CH_3_CN	21	83	69/31 (**7a**/**8a**)	5
2	CH_3_O*t*-Bu	47	93	72/24/4 (**7a**/**8a/9a**)	30
3	CHCl_3_	63	56	68/28/4 (**7a**/**8a/9a**)	51
4	ClCH_2_CH_2_Cl	63	55	70/30 (**7a**/**8a**)	51
5	C_6_H_5_Cl	16	69	75/25 (**7a/8a**)	50

^a^Isolated yield of all diastereoisomers after silica gel chromatography. ^b^Determined by ^1^H NMR analysis of the crude mixture. ^c^Determined by chiral HPLC analysis.

It is worth noting that bifunctional organocatalyst **VII** appears to be more effective in terms of diastereocontrol than previously employed Brønsted base/Lewis acid system TMG/ZnI_2_ giving four diastereoisomers instead [21].

Finally, the sulfa-Michael/nitroaldol cascade reaction was applied to other *trans*-β-methyl-β-nitrostyrenes under the optimized conditions (Table 3).

**Table 3:** Asymmetric sulfa-Michael/nitroaldol reaction of nitroalkenes **2** with 1,4-dithiane-2,5-diol (**4**) catalysed by amine thiourea **VII**.

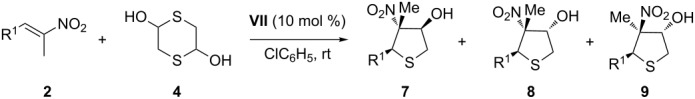					

entry	R^1^	time (h)	yield [%]^a^	dr^b^	ee **7** [%]^c^

1	Ph	16	69	75/25 (**7a**/**8a**)	50
2	4-ClC_6_H_4_	16	66	69/31 (**7b**/**8b**)	51
3	4-MeC_6_H_4_	40	76	74/26 (**7c**/**8c**)	42
4	2-naphthyl	45	90	66/26/8 (**7d**/**8d/9d**)	59

^a^Isolated yield of all diastereoisomers after silica gel chromatography. ^b^Determined by ^1^H NMR analysis of the crude mixture. ^c^Determined by chiral HPLC analysis.

Tetrahydrothiophenes, bearing three contiguous stereocenters, were isolated in good to high yield, moderate diastereoselectivity and up to 59% ee.

## Conclusion

In conclusion, we reported a diastereo- and enantioselective cascade sulfa-Michael/nitroaldol reaction of (*E*)-1-aryl-2-nitropropenes with 1,4-dithiane-2,5-diol. The process was catalyzed by an easily available amine thiourea to give 3,4,5-substituted tetrahydrothiophenes, bearing a quaternary stereocenter, in good yield and moderate enantiocontrol. It has been demonstrated that a simple bifunctional amine thiourea secures a more effective control of the diastereoselectivity than Brønsted base/Lewis acid systems. Data herein illustrated suggest that fine tuning of the bifunctional organocatalyst structure and reaction conditions will be required for further improvements of the challenging cascade process.

## Supporting Information

File 1 Experimental procedures, characterization data, NMR spectra of new compounds and HPLC traces of synthesized compounds, computational details.
