# Supplementary material for: Stereoselective amine-thiourea-catalysed sulfa-Michael/nitroaldol cascade approach to 3,4,5-substituted tetrahydrothiophenes bearing a quaternary stereocenter
Source: Beilstein J Org Chem. 2016 Apr 5;12:643–7. doi: 10.3762/bjoc.12.63 (PMC4901985; doi:10.3762/bjoc.12.63)
Supplement: File 1 — Experimental procedures, characterization data, NMR spectra of new compounds and HPLC traces of synthesized compounds, computational details. [file Beilstein_J_Org_Chem-12-643-s001.pdf]

Supporting Information

for

Stereoselective amine-thiourea-catalysed sulfa-Michael/nitroaldol cascade approach to 3,4,5-substituted tetrahydrothiophenes bearing a quaternary stereocenter

Sara Meninno, Chiara Volpe, Giorgio Della Sala, Amedeo Capobianco, and Alessandra Lattanzi*

Address: Dipartimento di Chimica e Biologia "A. Zambelli", Via Giovanni Paolo II, 84084, Fisciano, Italy

Email: Alessandra Lattanzi - lattanzi@unisa.it

*Corresponding author

Experimental procedures, characterization data, NMR spectra of new compounds and HPLC traces of synthesized compounds, computational details

Table of contents

General methods........S2

Experimental procedures and compounds characterization........S3

General procedure for the synthesis of catalyst **VIII**........S3

General procedure for the synthesis of racemic tetrahydrothiophenes........S4

General procedure for the asymmetric synthesis of tetrahydrothiophenes **5-11**........S4

Table S1........S10

NMR spectra........S11

Confirmation of the relative configuration of compounds 7a, 8a and 9a by NOESY and NOE........S33

HPLC chromatograms........S36

General methods

All reactions requiring dry or inert conditions were conducted in flame-dried glassware under a positive pressure of nitrogen. THF and DCM were freshly distilled prior to use respectively over metallic Na and calcium hydride and stored under nitrogen, all other solvents were dried over molecular sieves. Molecular sieves (Aldrich Molecular Sieves, 3 Å, 1.6 mm pellets) were activated under vacuum at 200 °C overnight.

Reactions were monitored by thin layer chromatography (TLC) on Macherey-Nagel pre-coated silica gel plates (0.25 mm) and visualized by UV light and, when necessary, by phosphomolybdic acid and ninhydrin staining solutions. Flash chromatography was performed on Merck silica gel (60, particle size: 0.040–0.063 mm). ^{1}H NMR and ^{13}C NMR spectra were recorded on Bruker Avance-400, Bruker Avance-300 or Bruker Avance-250 spectrometers in $CDCl_3$ as solvent at room temperature. NOE and NOESY spectra were recorded on Bruker Avance III HD 600 spectrometer in $CDCl_3$ as solvent. Chemical shifts for protons are reported using residual solvent protons (^{1}H NMR: δ = 7.26 ppm for $CDCl_3$) as internal standard. Carbon spectra were referenced to the shift of the ^{13}C signal of $CDCl_3$ (δ =77.0 ppm).

The following abbreviations are used to indicate the multiplicity in NMR spectra: s - singlet; d - doublet; t - triplet; q - quartet; dd – double doublet; m - multiplet; bs - broad singolet; bd – broad doublet.

Optical rotation of compounds was performed on a Jasco Dip-1000 digital polarimeter using the Na lamp (λ = 582 nm) and on a Jasco P-2000 digital polarimeter using WI (Tungsten-Halogen) lamp (λ = 589 nm). FTIR spectra were recorded as thin films on KBr plates using Bruker Vertex 70 spectrometer or Bruker Tensor 27 spectrometer and absorption maxima are reported in wavenumber (cm^{-1}). ESIMS was performed using a Bio-Q triple quadrupole mass spectrometer (Micromass, Manchester, UK) equipped with an electrospray ion source. Melting points were measured with a Stuart Model SMP 30 melting point apparatus or with an Electrothermal 9100 apparatus and are uncorrected. Petrol ether (PE) refers to light petroleum ether (boiling point 40–60 °C). Anhydrous toluene, chlorobenzene, methanol, acetonitrile and all starting materials (unless otherwise noted) were purchased from Aldrich and used as received.

The (*E*)-α,β-disubstituted nitroalkenes **2**, **3** were synthesized according to the literature.[1] Catalyst **I** was purchased from Aldrich and compound **V** from Strem Chemicals and used as received. Amine-

[1] (a) Kawai Y.; Inaba Y.; Tokitoh N. *Tetrahedron: Asymmetry*, **2001**, *12*, 309-318; (b) Fioravanti S.; Pellacani L.; Stabile S; Tardella P. A.; Ballini R. *Tetrahedron*, **1998**, *54*, 6169-6176; (c) Ballini R.; Castagnani R.; Petrini M. *J. Org. Chem.*, **1992**, *57*, 2160-2162; (d) Ballini R.; Palestini C. *Tetrahedron Lett.*, **1994**, *35*, 5731-5734.

thiourea **II** was synthesized according to the literature.[2] Catalysts **IV**,[3] **III**,[4] **VI**,[5] **VII**[6] are known compounds, they were prepared according to the literature. Enantiomeric excess of compounds **5**,**6**,**7**,**11** was determined by HPLC (Waters-Breeze 2487, UV dual λ absorbance detector and 1525 Binary HPLC Pump) using Daicel chiral columns.

Experimental procedures and compounds characterization

General procedure for the synthesis of catalyst VIII

Under a positive pressure of nitrogen, a flamed two necked round bottom flask was charged with anhydrous MeOH (500 μL) and (1*R*,2*R*)-(+)-1,2-diphenylethylenediamine (200 mg, 0.943 mmol). Subsequently, pivaladehyde (112 μL, 1.031 mmol) was added to the solution and the mixture was stirred for 1 h at room temperature. After this time, the reaction was cooled to 0 °C and $NaBH_4$ (143 mg, 3.77 mmol) was added. The resulting mixture was stirred at room temperature for 2 h.

After 2 h, the reaction mixture was transferred to a separatory funnel with ethyl acetate (20 mL) and H_2O. The organic layer was washed three times with H_2O (3 × 20 mL) and dried (Na_2SO_4). The ethyl acetate was removed in vacuo and the crude residue loaded onto a silica gel column and chromatographed (eluting from PE/ethyl acetate 80/20 to $CHCl_3$ to $CHCl_3$/MeOH 90:10) to afford (1*R*,2*R*)-N^1-neopentyl-1,2-diphenylethane-1,2-diamine (173 mg, 65% yield). Spectral data for this compound were consistent with those reported in the literature.[7] (1*R*,2*R*)-N^1-neopentyl-1,2-diphenylethane-1,2-diamine (173 mg, 0.61 mmol) was dissolved in anhydrous DCM (4 mL) in a flamed two necked round bottom flask under a positive pressure of nitrogen. 3,5-Bis(trifluoromethyl)phenyl isothiocyanate (134 μL, 0.73 mmol) was added and the reaction mixture was stirred at room temperature until completion, monitored by TLC (eluent PE/ethyl acetate 8:2). The product was purified by flash chromatography (eluent 10–50% Et_2O in PE) to give catalyst **VIII** (192 mg, 57% yield) as white solid.

[2] Vakulya, B.; Varga S.; Csámpai, A.; Soós T. *Org. Lett.* **2005**, *7*, 1967-1969.
[3] Yang, W.; Du, D.-M. *Org. Lett.* **2010**, *12*, 5450-5453.
[4] (a) Miyaji, R.; Asano K.; Matsubara, S. *Org. Lett.* **2013**, *15*, 3658-3661; (b) Wu, W.; Min, L.; Zhu, L.; Lee, C.-S. *Adv. Synth. Catal.* **2013**, *353*, 1135-1145.
[5] Liu, T.-Y.; Long, J.; Li, B.-J.; Jiang, L.; Li, R.; Wu, Y.; Ding, L.-S.; Chen, Y.-C. *Org. Biomol. Chem.* **2006**, *4*, 2097-2099.
[6] Meninno, S.; Croce, G.; Lattanzi, A. *Org. Lett.*, **2013**, *15*, 3436–3439.
[7] Wang, M.; Lin, L.; Shi, J.; Liu, X.; Kuang, Y.; Feng, X. *Chem. Eur. J.* **2011**, *17*, 2365-2368.

1-(3,5-Bis(trifluoromethyl)phenyl)-3-((1*R*,2*R*)-2-(neopentylamino)-1,2-diphenylethyl)thiourea (VIII)

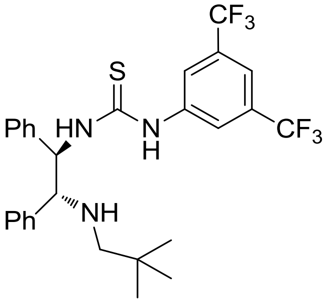

White solid, **m.p.** 74.5-75.9 °C. $[\alpha]_D^{28}$ = +36.2 (*c* 1.00, $CHCl_3$). **FTIR** ν_{max}(KBr)/cm^{-1} 2956, 1509, 1474, 1383, 1278, 1178, 1136, 887, 758, 701. **^{1}H NMR** ($CDCl_3$, 400 MHz): δ 7.77 (s, 2H), 7.73 (bs, 1H), 7.37-7.21 (m, 6H), 7.19-7.16 (m, 2H), 7.10-7.08 (m, 2H), 5.19 (bs,1H), 3.89 (bd, 1H, *J*= 5.2 Hz), 2.19-2.15 (m, 1H), 1.99-1.90 (m, 1H), 0.71 (bs, 9H).**^{13}C NMR** ($CDCl_3$, 100 MHz): δ 181.6, 139.6, 138.9, 132.9, 129.2, 128.9, 128.2, 127.5, 126.9, 125.1, 124.4, 121.7, 120.0, 68.6, 65.1, 59.7, 31.6, 27.6. **MS** (ESI$^+$ m/z): 554.6 [MH$^+$, 100%].

General procedure for the synthesis of racemic tetrahydrothiophenes

A sample vial was charged with nitroalkene (0.1 mmol), 1,4-dithiane-2,5-diol (**4**, 22.8 mg, 0.15 mmol), NEt_3 (2.8 μL, 0.02 mmol) and dry toluene (500 μL). The reaction was stirred at room temperature until completion, monitored by TLC (eluent PE/Et_2O 7/3). The racemic products were isolated (48–96%) by flash chromatography (eluting in gradient 10–30% of ethyl acetate in PE).

General procedure for the asymmetric synthesis of tetrahydrothiophenes 5–11

To a solution of nitroalkene **2** (0.1 mmol) in dry chlorobenzene (500 μL) or nitroalkene **1** or **3** in dry toluene (500 μL), 1,4-dithiane-2,5-diol **4** (22.8 mg, 0.15 mmol) and the catalyst **VII** (5.7 mg, 0.01 mmol) were added. The resulting mixture was stirred at room temperature until completion (TLC eluent PE/Et_2O 7/3). The diastereoisomeric ratio was determined by ^{1}H NMR analysis of the crude reaction mixture, after filtration of the crude reaction with CH_2Cl_2 onto flash silica gel to remove the poorly soluble 1,4-dithiane-2,5-diol. Then, the crude mixture of compound **5**–**6**, **7–9** and **10–12** was purified by flash chromatography (eluting in gradient 10–30% of ethyl acetate in PE) to isolate the major and minor diastereomers or their mixture (66–90%).

(3*S,4*R**,5*S**)-4-Nitro-5-phenyltetrahydrothiophen-3-ol (5) major diastereomer**

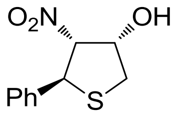

5

Data for this compound were consistent with those reported in the literature.[8] HPLC analysis with Chiralcel OD-H column, 90:10 *n*-hexane:2-propanol, 1 mL/min, 254 nm; minor enantiomer t_R = 14.6 min, major enantiomer t_R = 19.2 min.

(3*R,4*R**,5*S**)-4-Nitro-5-phenyltetrahydrothiophen-3-ol (6) minor diastereomer**

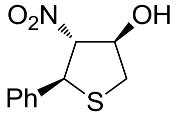

6

Data for this compound were consistent with those reported in the literature.[8] HPLC analysis with Chiralcel OD-H column, 90:10 *n*-hexane:2-propanol, 1 mL/min, 254 nm; minor enantiomer t_R = 11.0 min, major enantiomer t_R = 9.3 min.

(3*R,4*R**,5*S**)-4-Methyl-4-nitro-5-phenyltetrahydrothiophen-3-ol (7a)**

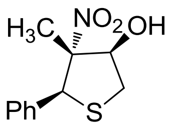

7a

White solid, **m.p.** 92.3-95.8°C. **$[\alpha]_D^{19}$** = -49.0 (c 0.50, $CHCl_3$), *ee* = 50%. **FTIR** ν_{max}(KBr)/cm^{-1} 3447, 2921, 1542, 1452, 1385, 1348, 1082, 1032, 749, 701. **^{1}H NMR** ($CDCl_3$, 400 MHz): δ 7.39-7.29 (m, 5H), 5.20 (s, 1H), 5.20-5.12 (m, 1H), 3.23 (dd, 1H, J_2=7.5 Hz, J_1=10.3 Hz), 2.87 (dd, 1H, J_1=J_2=10.0 Hz), 2.56 (d, 1H, J=5.0 Hz), 1.43 (s, 3H). **^{13}C NMR** ($CDCl_3$, 100 MHz): δ 134.2, 129.4, 129.2, 128.7, 98.4, 78.8, 53.2, 31.4, 11.5. **MS** (ESI^+ m/z): 278.2 [MK^+, 97%]. HPLC analysis with Chiralpak IE-3 column, 90:10 *n*-hexane:2-propanol, 0.7 mL/min, 220 nm; minor enantiomer t_R = 14.6 min, major enantiomer t_R = 11.7 min.

---

[8] (a) O' Connor C. J.; Roydhouse M. D.; Przybyl A. M.; Wall M. D.; Southern J. M. *J. Org. Chem.* **2010**, *75*, 2534–2538; (b) Xu C.; Du J.; Ma L.; Li G.; Tao M.; Zhang W. *Tetrahedron* **2013**, *69*, 4749-4757.

(3*S**,4*R**,5*S**)-4-Methyl-4-nitro-5-phenyltetrahydrothiophen-3-ol (8a) and (3*S,4*S**,5*S**)-4-methyl-4-nitro-5-phenyltetrahydrothiophen-3-ol (9a) mixture**

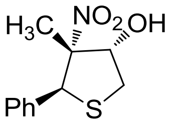

8a
major

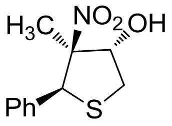

9a
minor

Colorless waxy solid. **^{1}H NMR** ($CDCl_3$, 400 MHz): δ 7.54 (d, 2H major), 7.39-7.27 (m, 3H major + 5H minor), 5.44 (s, 1H, major), 5.30 (dd, 1H, J_1=J_2=8.3 Hz, minor), 4.57 (dd, 1H, J_1=J_2=4.5 Hz, major), 4.47 (s, 1H, minor), 3.44 (dd, 1H, J_2=7.5 Hz, J_1=10.5 Hz, minor), 3.38 (dd, 1H, J_2=5.2 Hz, J_1=11.5 Hz, major), 3.07 (bs, 1H, major), 3.00 (dd, 1H, J_2=4.3 Hz, J_1=11.5 Hz, major), 2.88 (dd, 1H, J_1=J_2=10.0 Hz, minor), 2.46 (bs, 1H, minor), 1.95 (s, 3H, minor), 1.47 (s, 3H, major).**^{13}C NMR** ($CDCl_3$, 100 MHz): δ 137.6 (minor), 135.2 (major), 130.3, 129.4, 128.9, 128.8, 128.6, 99.3 (major), 99.0 (minor), 80.5 (major), 73.8 (minor), 55.9 (minor), 52.0 (major), 34.0 (major), 31.4 (minor), 20.1 (major), 19.7 (minor). **MS** (ESI^+ m/z): 261.6 [MNa^+, 100%].

(3*S**,4*R**,5*S**)-4-Ethyl-4-nitro-5-phenyltetrahydrothiophen-3-ol (11) major diastereomer[9]

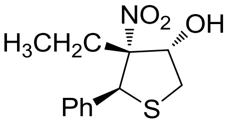

11

Colorless waxy solid. **^{1}H NMR** ($CDCl_3$, 400 MHz): δ 7.60-7.50 (m, 2H), 7.39-7.28 (m, 3H), 5.27 (s, 1H), 4.75-4.65 (m, 1H), 3.40 (d, 1H, *J*=8.5 Hz), 3.35 (dd, 1H, J_2=5.6 Hz, J_1=11.3 Hz,), 2.93 (dd, 1H, J_2=6.0 Hz, J_1=11.3 Hz), 2.10-1.90 (m, 1H), 1.81-1.70 (m, 1H), 0.73 (t, 3H, *J*=7.4 Hz).**^{13}C NMR** ($CDCl_3$, 100 MHz): δ 136.2, 130.4, 128.8, 128.6, 102.3, 78.4, 52.4, 33.1, 26.9, 8.1. **MS** (ESI^+ m/z): 276.8 [MNa^+, 11%], 292.5 [MK^+, 15%]. HPLC analysis with Chiralpak IA-3 column, 95:5 *n*-hexane:2-propanol, 0.8 mL/min, 220 nm; minor enantiomer t_R = 29.5 min, major enantiomer t_R = 24.5 min.

[9] The relative configuration of this compound has been assigned on the similarity of chemical shifts observed in the ^{1}H-NMR spectra with those of compound **8a**.

(3*R,4*R**,5*S**)-4-Ethyl-4-nitro-5-phenyltetrahydrothiophen-3-ol (10) minor diastereomer**[10]

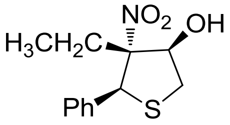

10

Colorless waxy solid. **FTIR** ν_{max}(KBr)/cm^{-1} 3454, 2943, 1539, 1454, 1364, 1336, 1160, 1079, 1024, 830, 747, 701. **^{1}H NMR** ($CDCl_3$, 400 MHz): δ 7.60-7.50 (m, 2H), 7.39-7.28 (m, 3H), 5.20-5.13 (m, 1H), 5.12 (s, 1H), 3.35 (dd, 1H, J_2=4.8 Hz, J_1=12.0 Hz), 3.12 (dd, 1H, J_2=4.0 Hz, J_1=12.0 Hz), 2.52 (d, 1H, J=7.2 Hz), 1.89-1.69 (m, 2H), 0.75 (t, 3H, J=7.3 Hz).**^{13}C NMR** ($CDCl_3$, 100 MHz): δ 136.2, 130.5, 128.8, 128.7, 104.5, 78.4, 56.4, 36.7, 25.9, 9.4. **MS** (ESI$^+$ m/z): 292.5 [MK$^+$, 15%].

(3*R,4*R**,5*S**)-5-(4-Chlorophenyl)-4-methyl-4-nitrotetrahydrothiophen-3-ol (7b)**

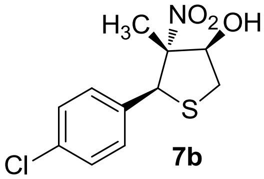

7b

White solid. **m.p.** 88.6-94 °C. **$[\alpha]_D^{20}$** = -25.6 (c 0.60, $CHCl_3$), *ee* = 51%. **FTIR** ν_{max}(KBr)/cm^{-1} 3447, 1635, 1539, 1490, 1349, 1087. **^{1}H NMR** ($CDCl_3$, 400 MHz): δ 7.34-7.18 (m, 4H), 5.15 (s, 1H) overlapped with 5.18-5.10 (m, 1H), 3.23 (dd, 1H, J_2=7.5 Hz, J_1=10.5 Hz), 2.87 (dd, 1H, J_1=J_2=10.3 Hz), 2.48 (bs, 1H), 1.42 (s, 3H). **^{13}C NMR** ($CDCl_3$, 62.5 MHz): δ 134.8, 132.6, 130.5, 128.7, 97.7, 78.3, 52.1, 31.1, 11.4. **MS** (ESI$^+$ m/z): 312.0 [MK$^+$, 20%]. HPLC analysis with Chiralpak IE-3 column, 90:10 *n*-hexane:2-propanol, 0.7 mL/min, 254 nm; minor enantiomer t_R = 25.6 min, major enantiomer t_R = 9.7 min.

[10] The relative configuration of this compound has been assigned on the similarity of chemical shifts observed in the ^{1}H-NMR spectra with those of compound **7a**.

(3*S**,4*R**,5*S**)-5-(4-Chlorophenyl)-4-methyl-4-nitrotetrahydrothiophen-3-ol (8b)

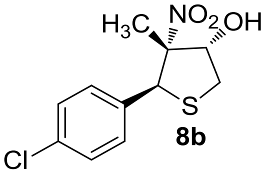

8b

The minor diastereomer **8b** was recovered by flash chromatography in a mixture with the major diasteromer **7b**. **^{1}H NMR** ($CDCl_3$, 400 MHz): δ 7.51 (d, 2H, *J*= 8.5 Hz), 7.32 (d, 2H, *J*= 8.5 Hz), 5.43 (s, 1H), 4.62-4.58 (m, 1H), 3.37 (dd, 1H, J_2=4.8 Hz, J_1=11.6 Hz), 2.98 (dd, 1H, J_2=3.5 Hz, J_1=11.7 Hz), 1.48 (s, 3H). **^{13}C NMR** ($CDCl_3$, 62.5 MHz): δ 134.4, 133.2, 131.5, 128.5, 98.8, 80.4, 50.7, 29.7, 19.6.

(3*R**,4*R**,5*S**)-4-Methyl-4-nitro-5-(*p*-tolyl)tetrahydrothiophen-3-ol (7c)

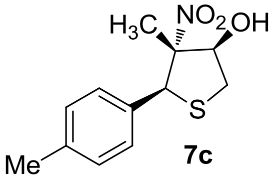

7c

Pale yellow solid. **m.p.** 75.8-80.2 °C. **$[\alpha]_D^{21}$** = -16.8 (c 0.64, $CHCl_3$), *ee* = 42%. **FTIR** ν_{max}(KBr)/cm^{-1} 3441, 1637, 1541, 1514, 1348, 1085. **^{1}H NMR** ($CDCl_3$, 300 MHz): δ 7.23 (d, 2H, *J*= 8.0 Hz), 7.13 (d, 2H, *J*=8.0 Hz), 5.15 (s, 1H) overlapped with 5.20-5.10 (m, 1H), 3.22 (dd, 1H, J_2=7.5 Hz, J_1=10.5 Hz), 2.85 (dd, 1H, J_1=J_2=10.2 Hz), 2.58 (d, 1H, J=5.4 Hz), 2.33 (s, 3H), 1.43 (s, 3H). **^{13}C NMR** ($CDCl_3$, 75 MHz): δ 138.8, 130.8, 129.1, 129.0, 98.0, 78.4, 52.7, 31.1, 21.1, 11.2.
MS (ESI$^+$ m/z): 252.8 [M$^+$, 27%], 293.3 [MK$^+$, 41%]. HPLC analysis with Chiralpak IE-3 column, 90:10 *n*-hexane:2-propanol, 0.7 mL/min, 220 nm; minor enantiomer t_R = 17.8 min, major enantiomer t_R = 11.1 min.

(3*S**,4*R**,5*S**)-4-Methyl-4-nitro-5-(*p*-tolyl)tetrahydrothiophen-3-ol (8c)

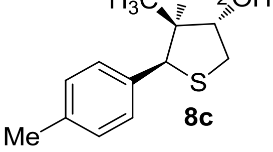

8c

The minor diastereomer **8c** was recovered by flash chromatography in a mixture with the major diastereomer **7c**. **^{1}H NMR** ($CDCl_3$, 400 MHz): δ 7.41 (d, 2H, *J*= 7.5 Hz), 7.15 (d, 2H, *J*= 7.5 Hz), 5.39 (s, 1H), 4.58-4.52 (m, 1H), 3.37 (dd, 1H, J_2=4.4 Hz, J_1=11.6 Hz), 2.98 (dd, 1H, J_2=4.4 Hz,

J_1=11.6 Hz), 2.34 (s, 3H), 1.94 (d, 1H, *J*= 0.9 Hz), 1.46 (s, 3H). ^{13}**C NMR** ($CDCl_3$, 100 MHz): *δ* 138.4, 131.9, 129.8, 129.1, 98.9, 80.1, 51.6, 33.7, 21.1, 19.8.

(3*R,4*R**,5*S**)-4-Methyl-5-(naphthalen-2-yl)-4-nitrotetrahydrothiophen-3-ol (7d)**

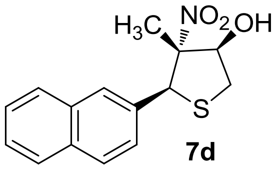

7d

White solid. **m.p.** 100.8-102.7 °C. **[α]$_D$^{20}** = -15.3 (c 0.17, $CHCl_3$), *ee* = 57%. **FTIR** ν_{max}(KBr)/cm^{-1} 3455, 1635, 1539, 1385, 1350, 1081. ^{1}**H NMR** ($CDCl_3$, 300 MHz): *δ* 7.90-7.77 (m, 4H), 7.54-7.47 (m, 2H), 7.43 (d, 1H, *J*= 8.6 Hz), 5.38 (s, 1H), 5.25-5.17 (m, 1H), 3.28 (dd, 1H, J_2=7.5 Hz, J_1=10.4 Hz), 2.93 (dd, 1H, J_1=J_2=10.3 Hz), 2.68 (bs, 1H), 1.47 (s, 3H). ^{13}**C NMR** ($CDCl_3$, 100 MHz): *δ* 133.4, 132.9, 131.4, 128.7, 128.2, 128.1, 127.6, 126.6, 126.5, 126.4, 98.2, 78.6, 53.0, 31.3, 11.5. **MS** (ESI$^+$ m/z): 312.0 [MNa$^+$, 37%], 328.0 [MK$^+$, 100%]. HPLC analysis with Chiralpak IE-3 column, 90:10 *n*-hexane:2-propanol, 0.7 mL/min, 254 nm; minor enantiomer t_R = 17.1 min, major enantiomer t_R = 12.2 min.

(3*S,4*R**,5*S**)-4-Methyl-5-(naphthalen-2-yl)-4-nitrotetrahydrothiophen-3-ol (8d) and (3*S**,4*S**,5*S**)-4-methyl-5-(naphthalen-2-yl)-4-nitrotetrahydrothiophen-3-ol (9d) mixture**

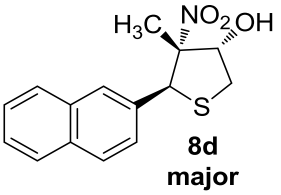

8d
major

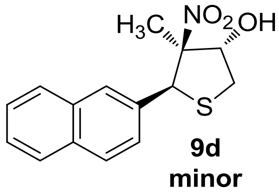

9d
minor

Pale yellow waxy solid. ^{1}**H NMR** ($CDCl_3$, 250 MHz): *δ* 8.04 (s, 1H major), 7.89-7.00 (m, 6H major + 7H minor), 5.62 (s, 1H, major), 5.45-5.39 (m, 1H, minor), 4.67-4.60 (m, 1H minor + 1H major), 3.51 (dd, 1H, J_2=7.3 Hz, J_1=10.4 Hz, minor), 3.43 (dd, 1H, J_2=4.9 Hz, J_1=11.3 Hz, major), 3.04 (dd, 1H, J_2=4.2 Hz, J_1=11.6 Hz, major), 1.99 (s, 3H, minor), 1.50 (s, 3H, major).^{13}**C NMR** ($CDCl_3$, 62.5 MHz): *δ* 134.7, 133.2, 132.4, 129.5, 128.8, 128.1, 128.0, 127.3, 126.6, 126.4, 126.2, 99.1 (major), 98.8 (minor), 80.4 (major), 73.7 (minor), 55.8 (minor), 51.9 (major), 33.9 (major), 29.7 (minor), 19.9 (major), 19.5 (minor).

Table S1. Predicted relative Gibbs free energies (kcal/mol, P = 1.0 atm, T = 298.15 K) of **8a**, **9a**, **14** in reference to **7a**, and **11**, **12**, **13** in reference to **10**.

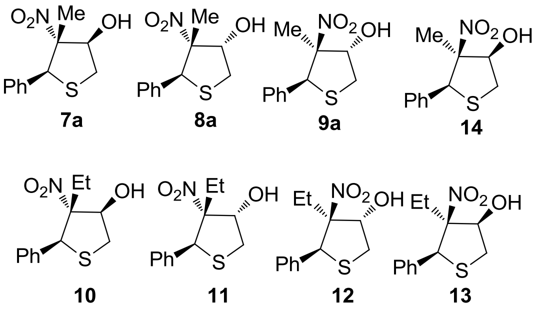

	Gibbs free energies (kcal/mol)
**8a**	+0.7
**9a**	+0.7
**14**	+1.2

	Gibbs free energies (kcal/mol)
**11**	–0.1
**12**	–0.1
**13**	+0.7

Computations (Table S1) on compound 4-methyl-4-nitro-5-phenyltetrahydrothiophen-3-ol find 7**a** as the most stable diastereomer among compounds **7a**, **8a**, **9a** and **14**. Compounds **8a** and **9a** lie at slightly higher free energy, with **14** being the least accessible diastereomer. Although small free energy differences between diastereomers are predicted, theoretical results are in line with the experimental observation that 7**a** is the most abundant product and **14** is not obtained at all, thus suggesting that the reaction may proceed under thermodynamic control. For compound 4-ethyl-4-nitro-5-phenyltetrahydrothiophen-3-ol **10**, **11** and **12** isomers should be equally populated according to DFT computations, but relative free energies are too close to draw any realistic conclusion about relative stability. By contrast, it is sound and worth to note that the diastereomers of compound 4-ethyl-4-nitro-5-phenyltetrahydrothiophen-3-ol span a narrower free energy range than that of 4-methyl-4-nitro-5-phenyltetrahydrothiophen-3-ol , which – under the hypothesis of thermodynamic control – is again in line with the lower diastereomeric ratio observed.

Computational details

DFT computations were carried out by using the Gaussian program.[11] The M06-2X functional in conjunction with the 6-31+G(d,p) basis set was used for geometry optimizations. Solvation effects (chlorobenzene) were included in all computations by the polarizable continuum model. [12]

[11] Gaussian 09, Revision D.01, M. J. Frisch, *et al*, Gaussian, Inc., Wallingford CT, **2009**.
[12] Miertuš S.; Scrocco E.; Tomasi J. *Chem. Phys.* **1981**, *55*, 117-129.

NMR Spectra

Compound **VIII** ^1H NMR in $CDCl_3$ (400 MHz)

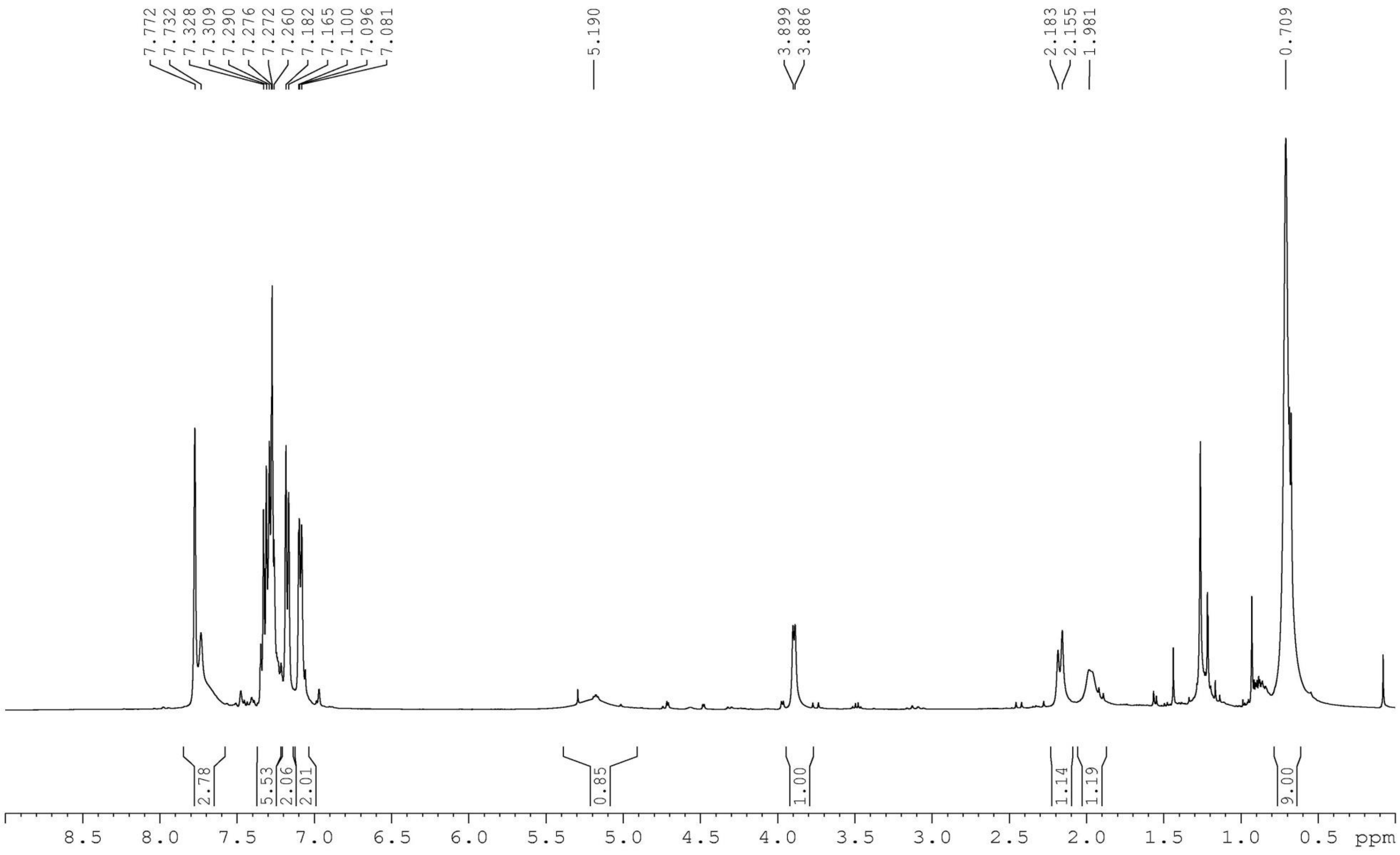

Compound **VIII** ^{13}C NMR in $CDCl_3$ (100 MHz)

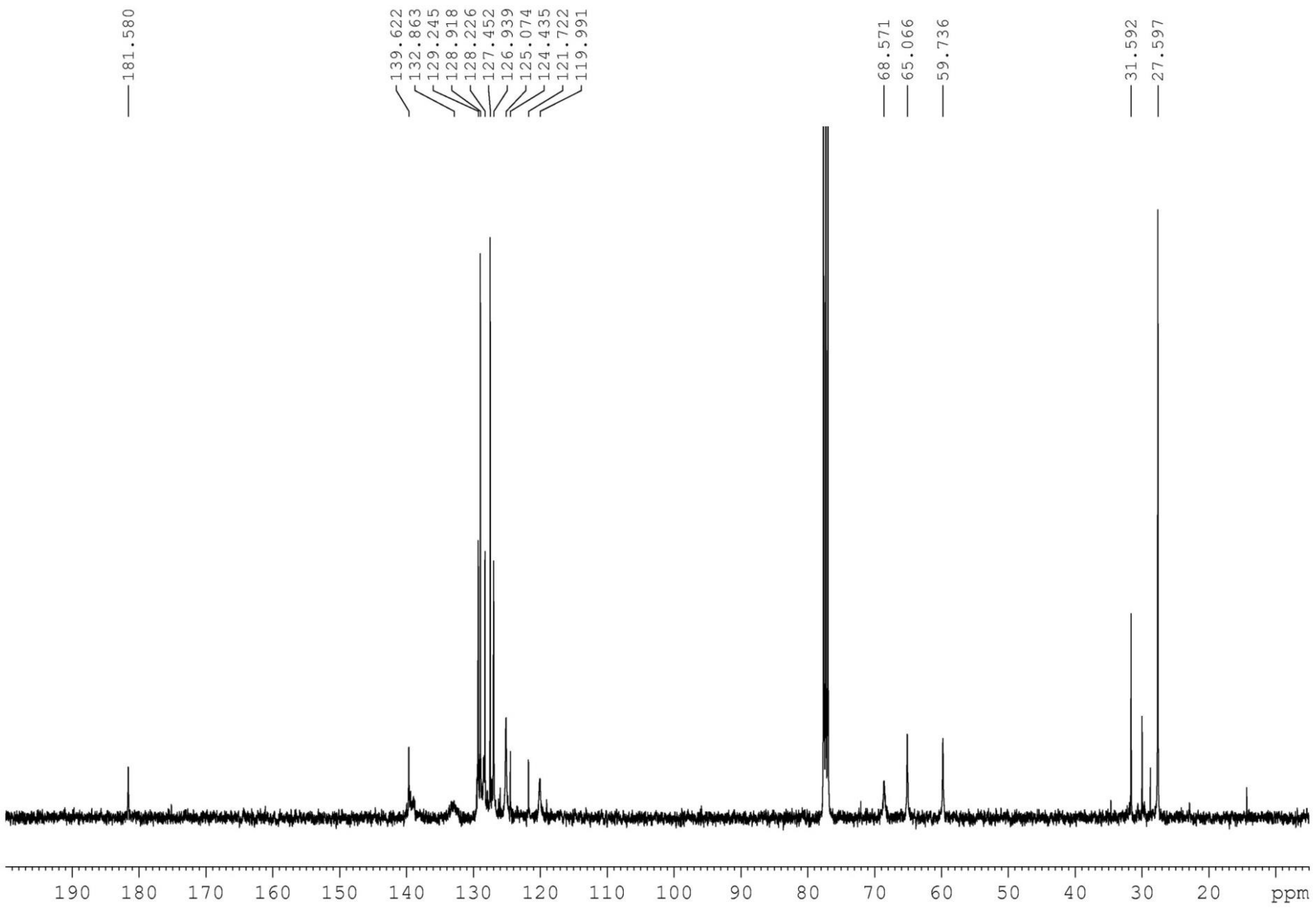

Compound **7a** ^{1}H NMR in $CDCl_3$ (400 MHz)

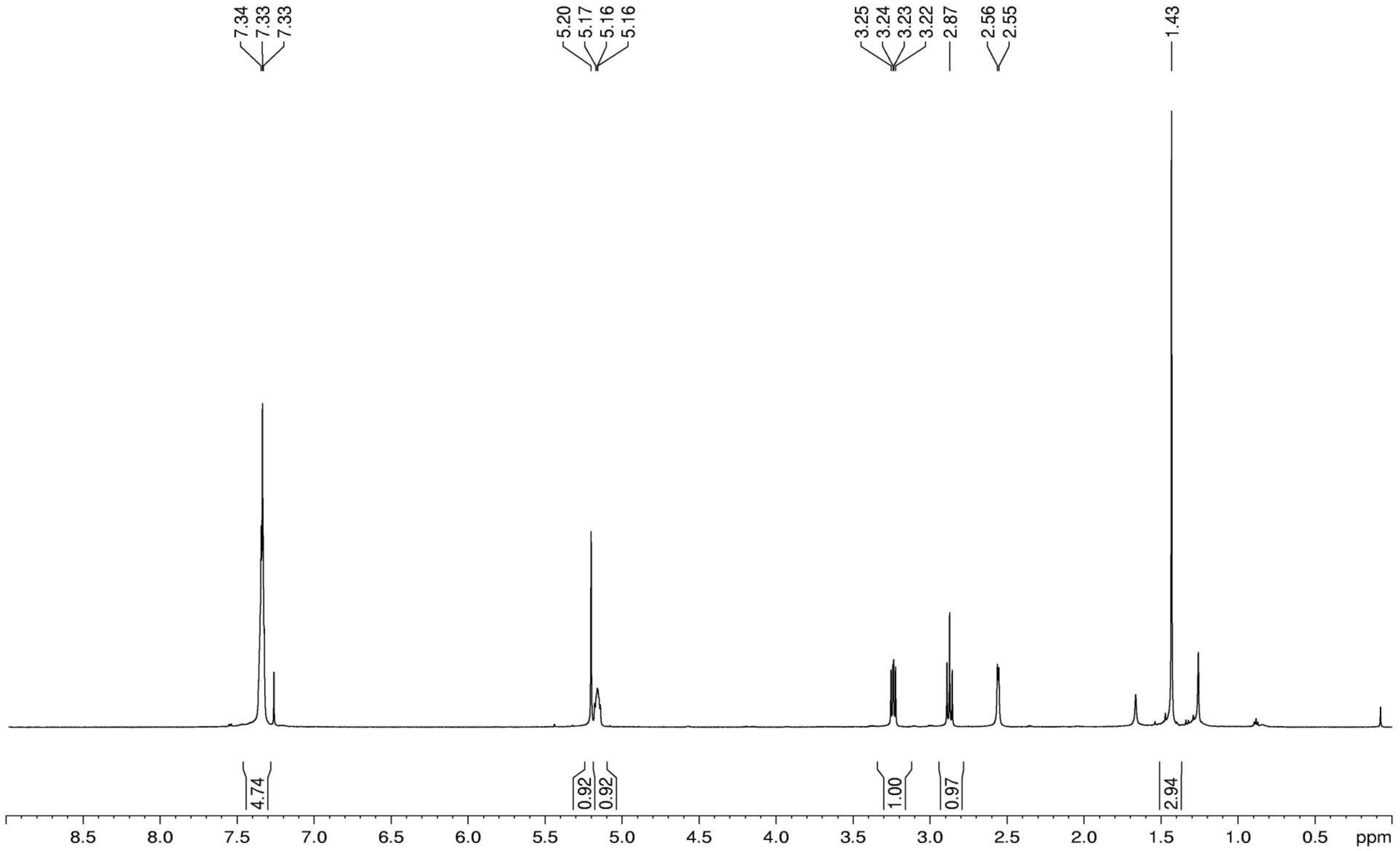

Compound **7a** ^{13}C NMR in $CDCl_3$ (100 MHz)

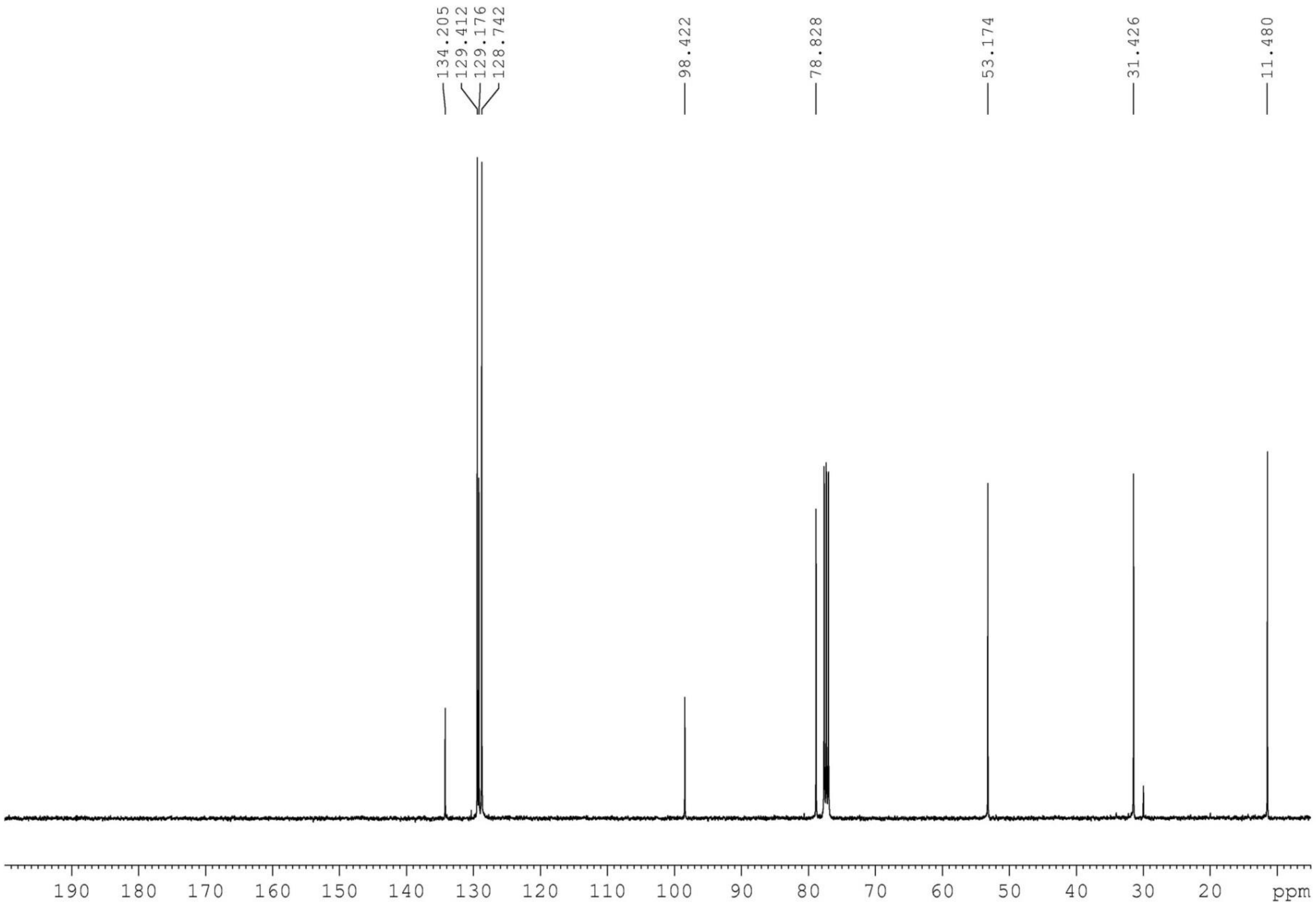

Mixture of compounds **8a** and **9a** ^{1}H NMR in $CDCl_3$ (400 MHz)

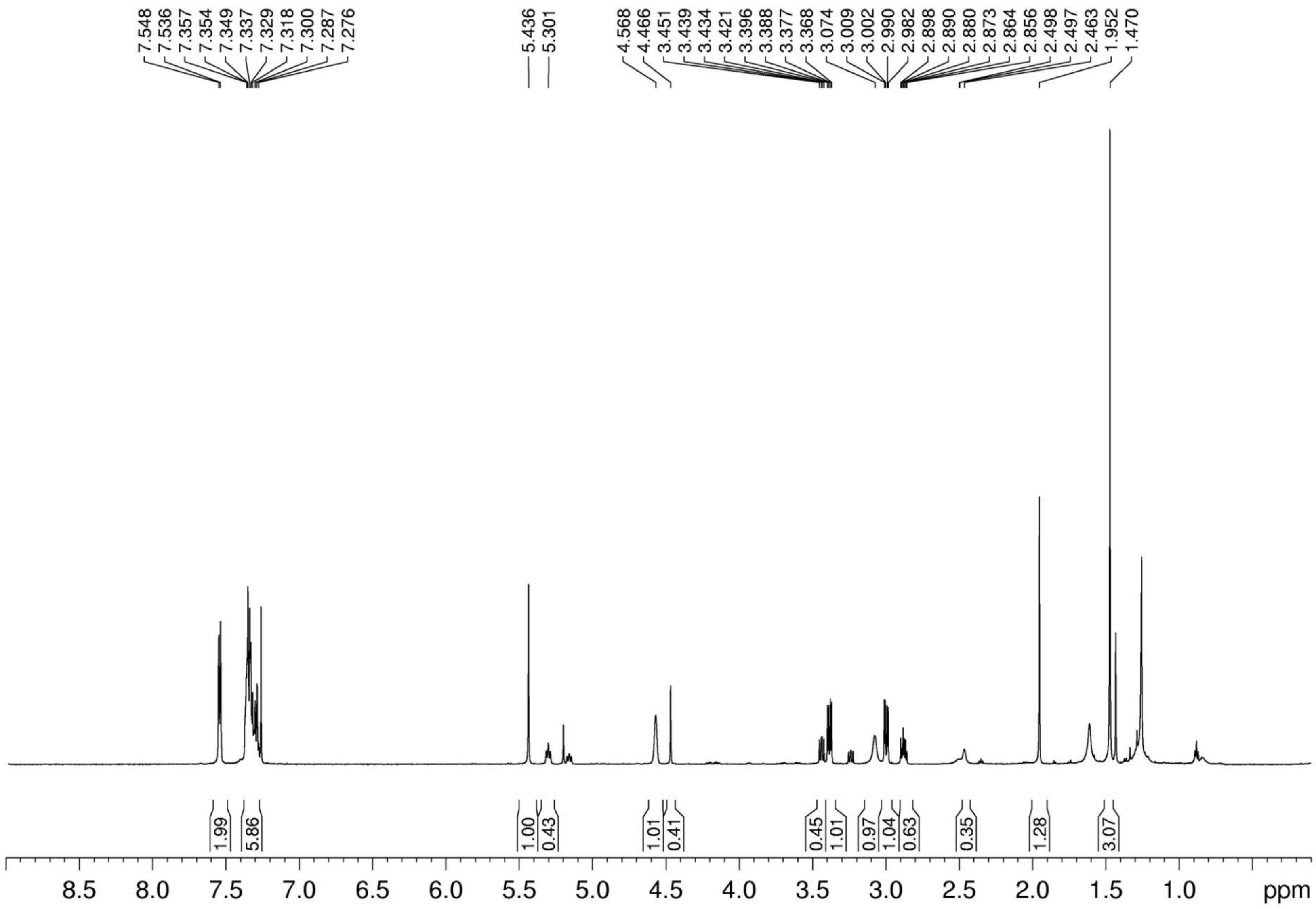

Mixture of compounds **8a** and **9a** ^{13}C NMR in $CDCl_3$ (100 MHz)

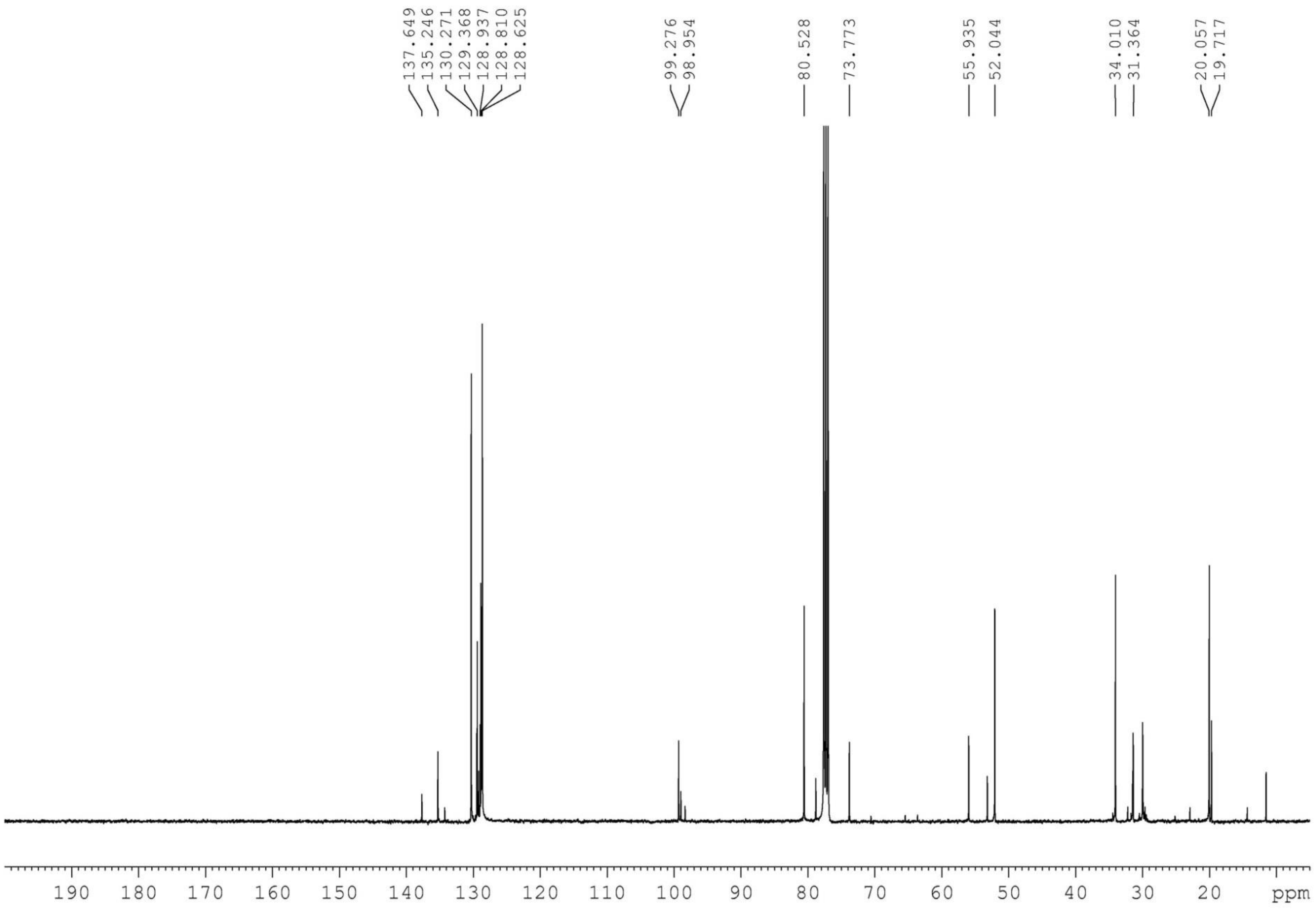

Compound **11** ^{1}H NMR in $CDCl_3$ (400 MHz)

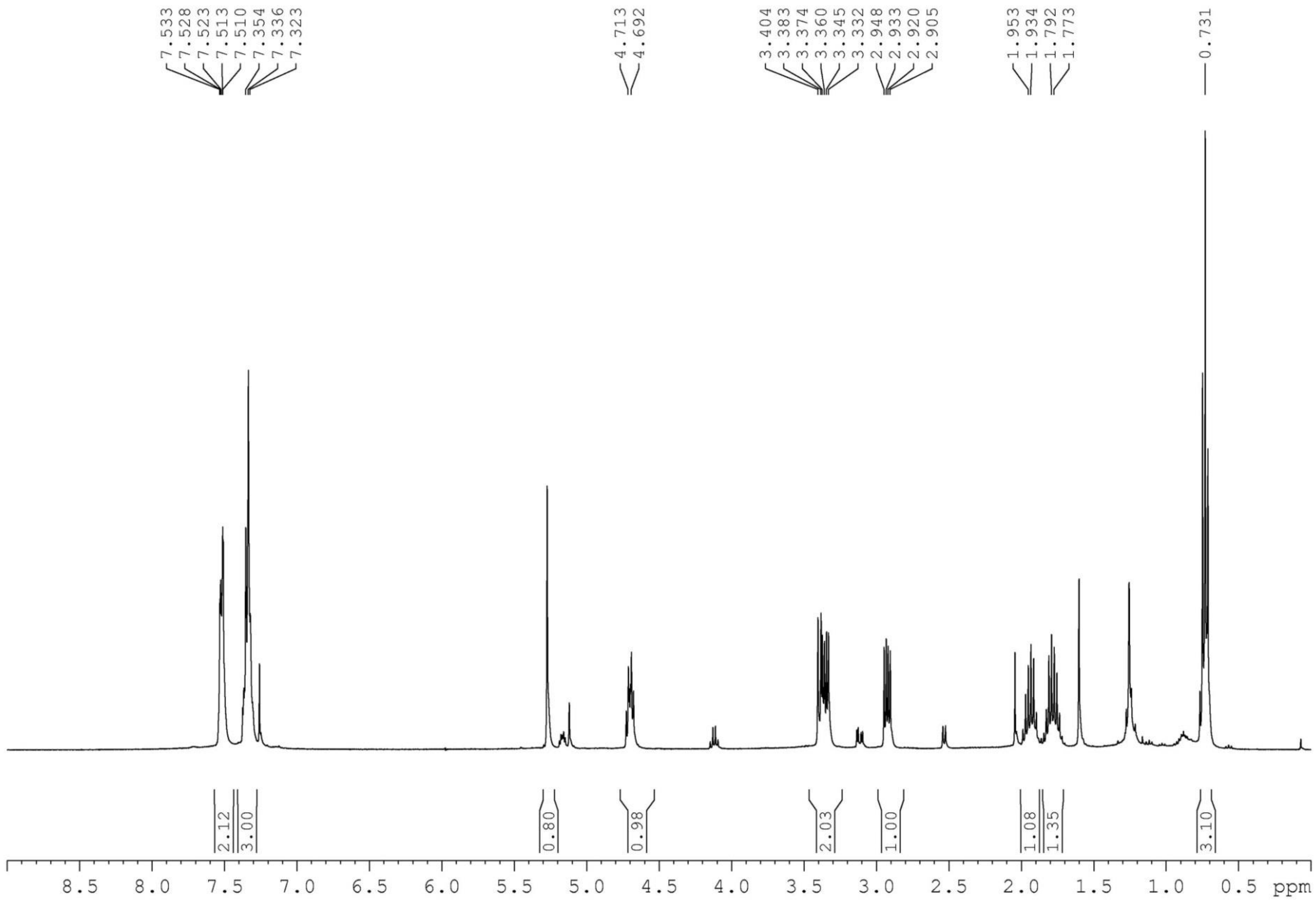

Compound **11** ^{13}C NMR in $CDCl_3$ (100 MHz)

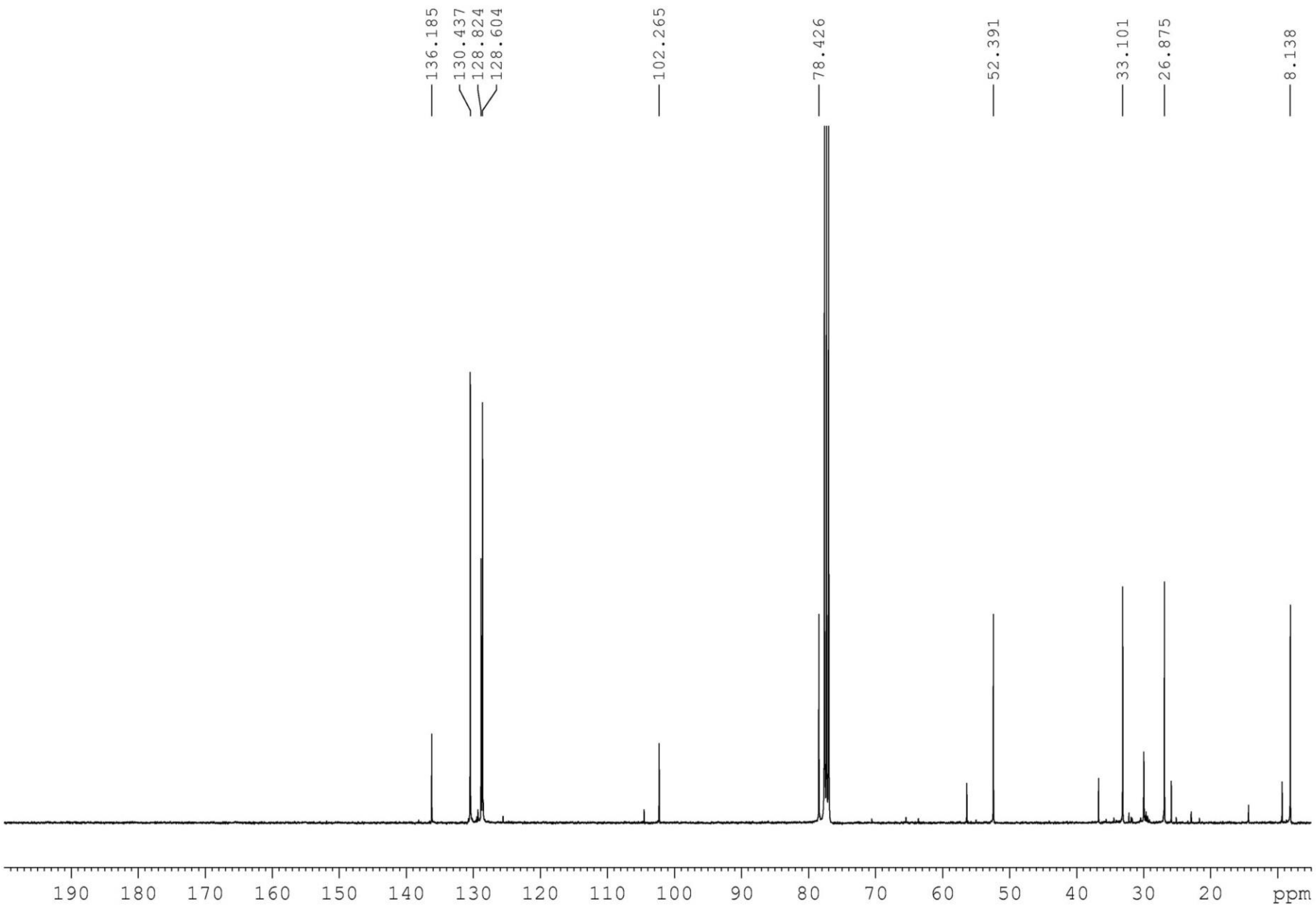

Compound **10** ^{1}H NMR in $CDCl_3$ (400 MHz)

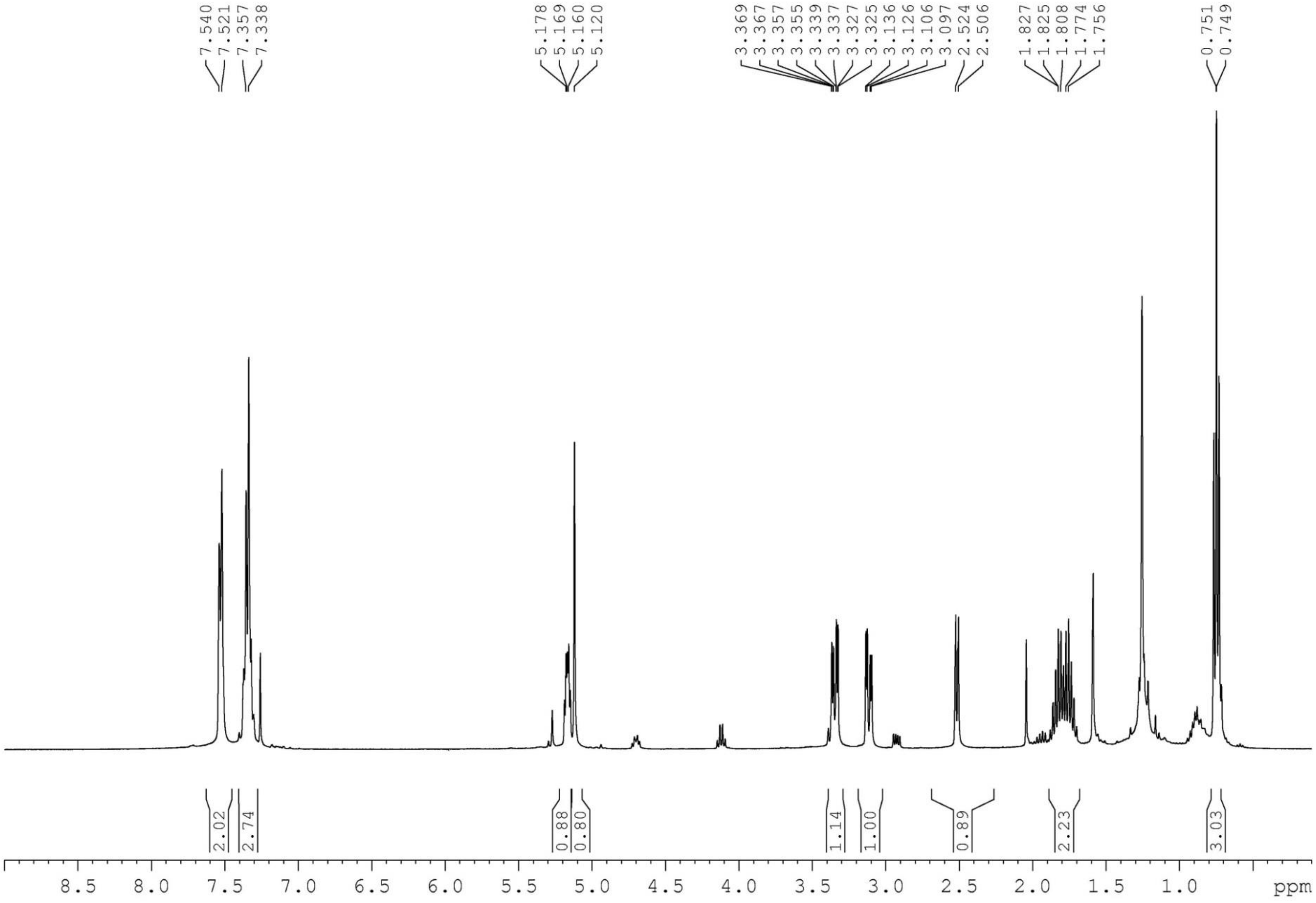

Compound **10** ^{13}C NMR in $CDCl_3$ (100 MHz)

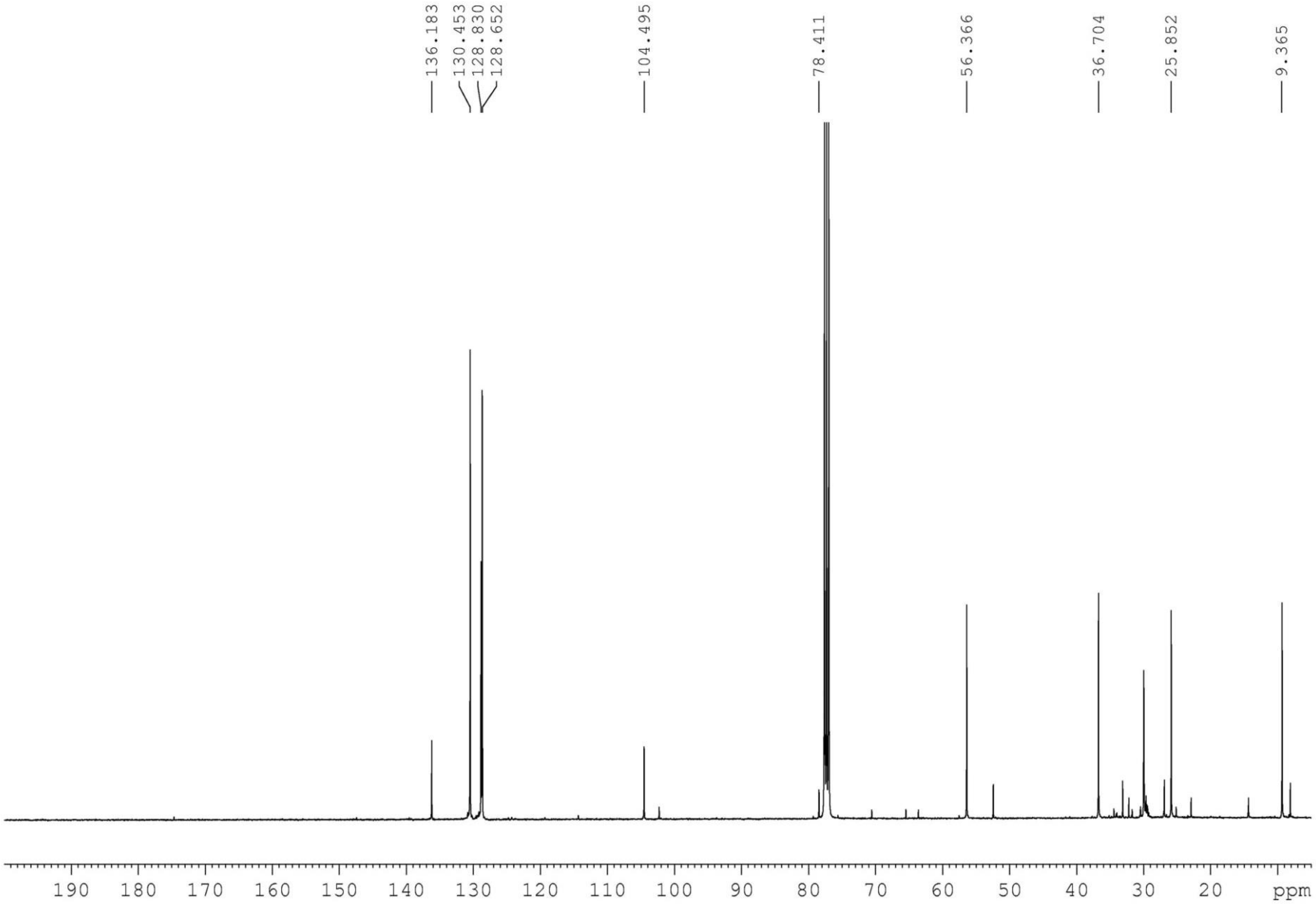

Compound **7b** ^{1}H NMR in $CDCl_3$ (400 MHz)

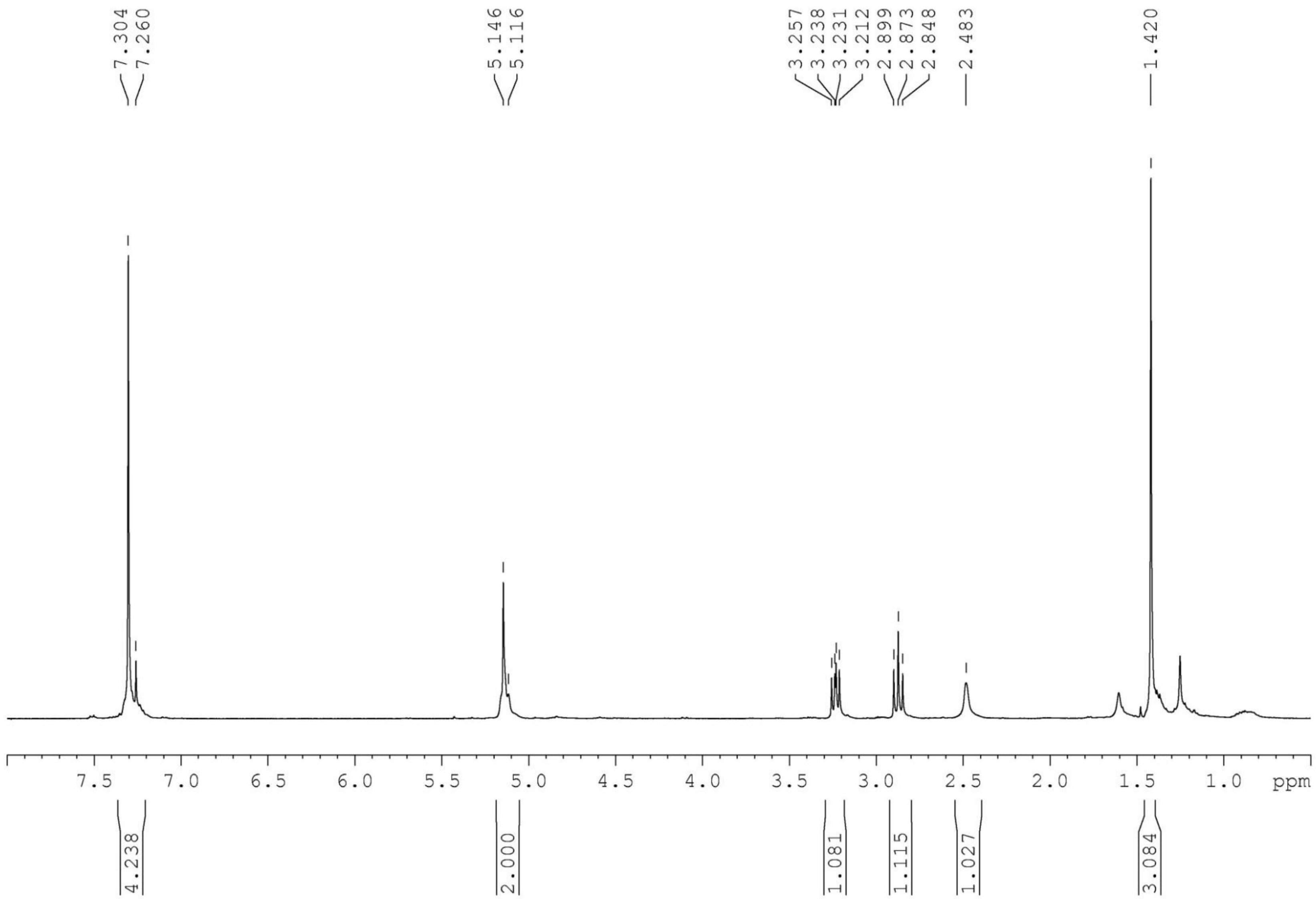

Compound **7b** ^{13}C NMR in $CDCl_3$ (62.5 MHz)

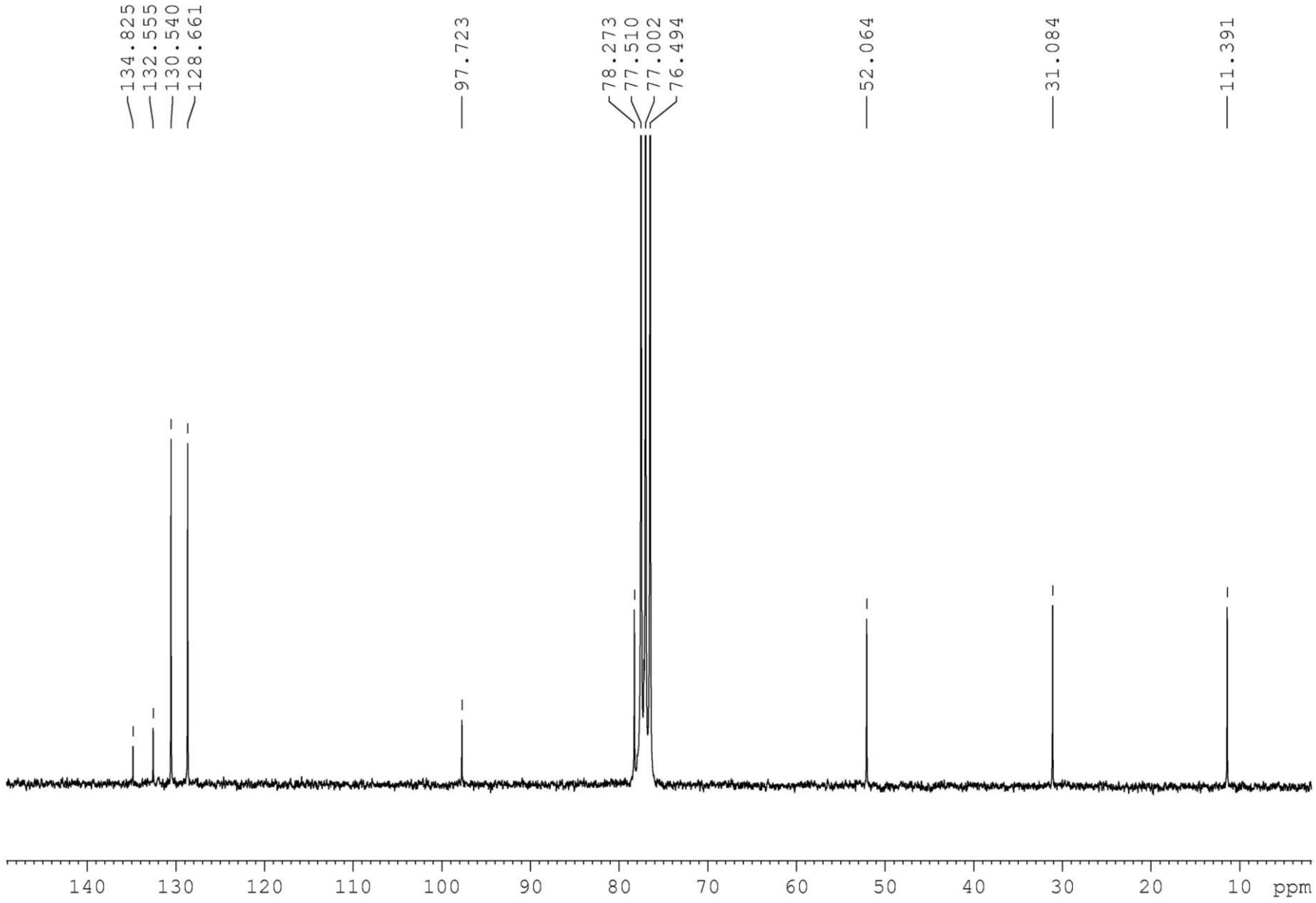

Compound **8b** ^{1}H NMR in $CDCl_3$ (400 MHz)

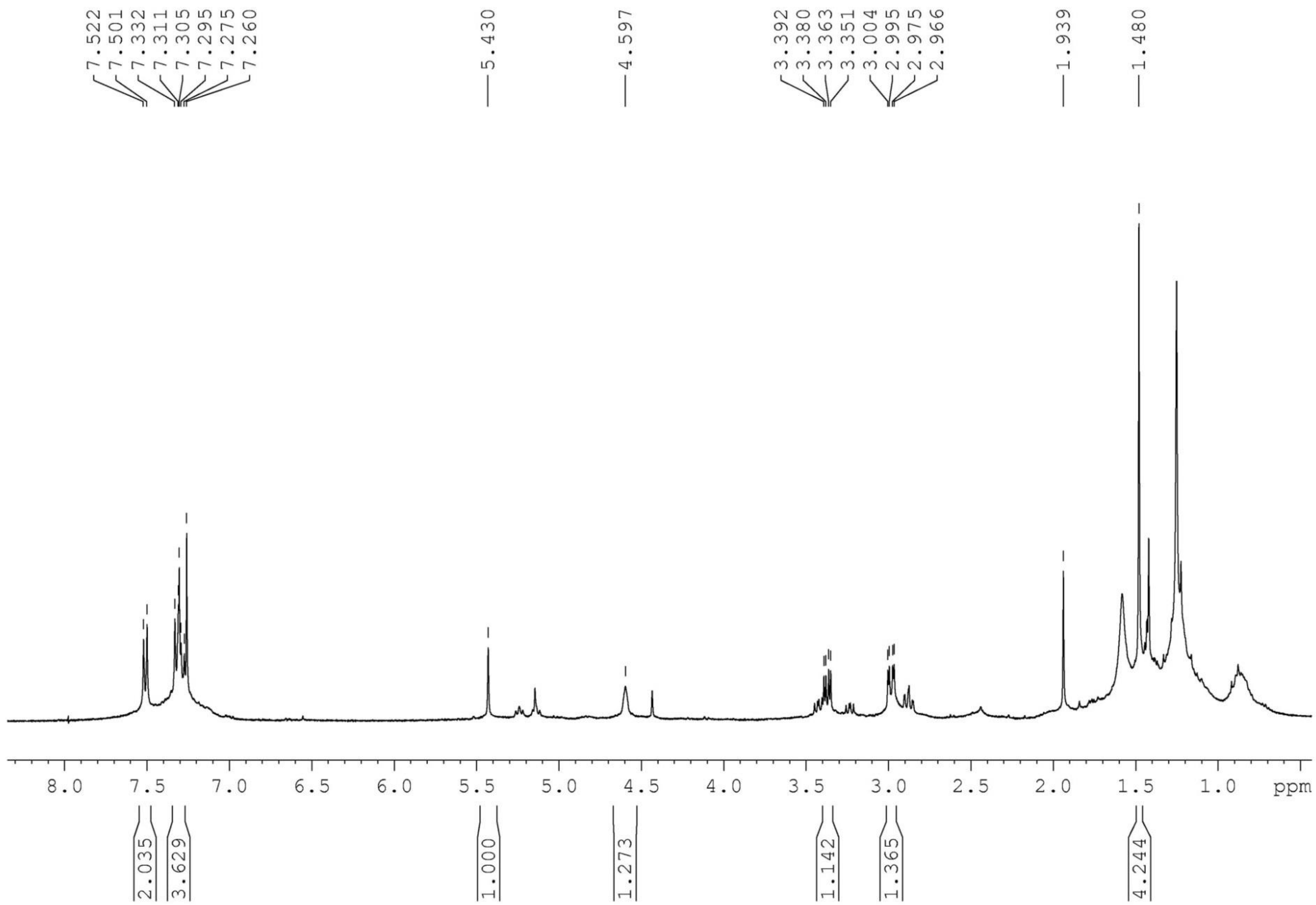

Compound **8b** ^{13}C NMR in $CDCl_3$ (62.5 MHz)

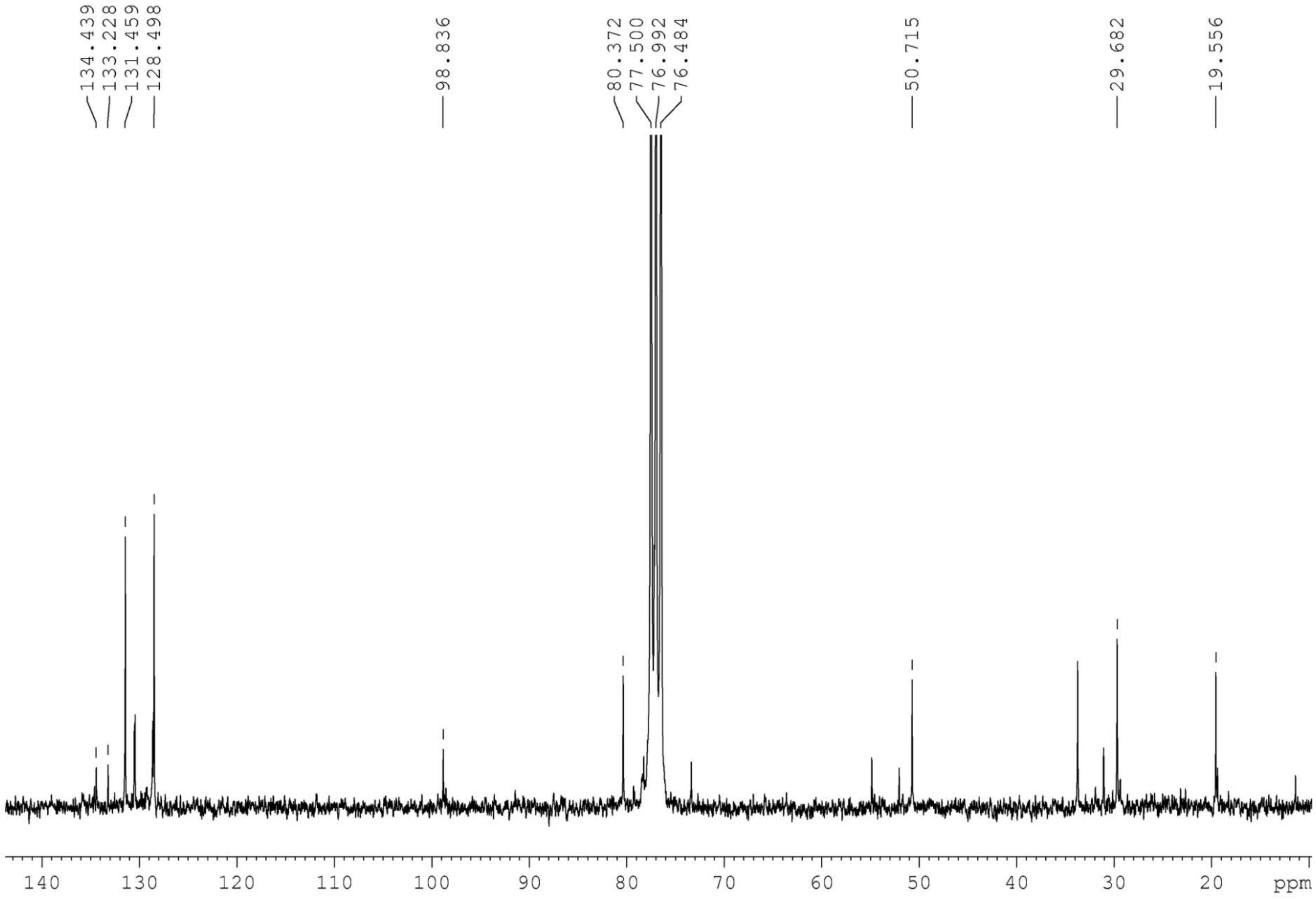

Compound **7c** ^{1}H NMR in $CDCl_3$ (300 MHz)

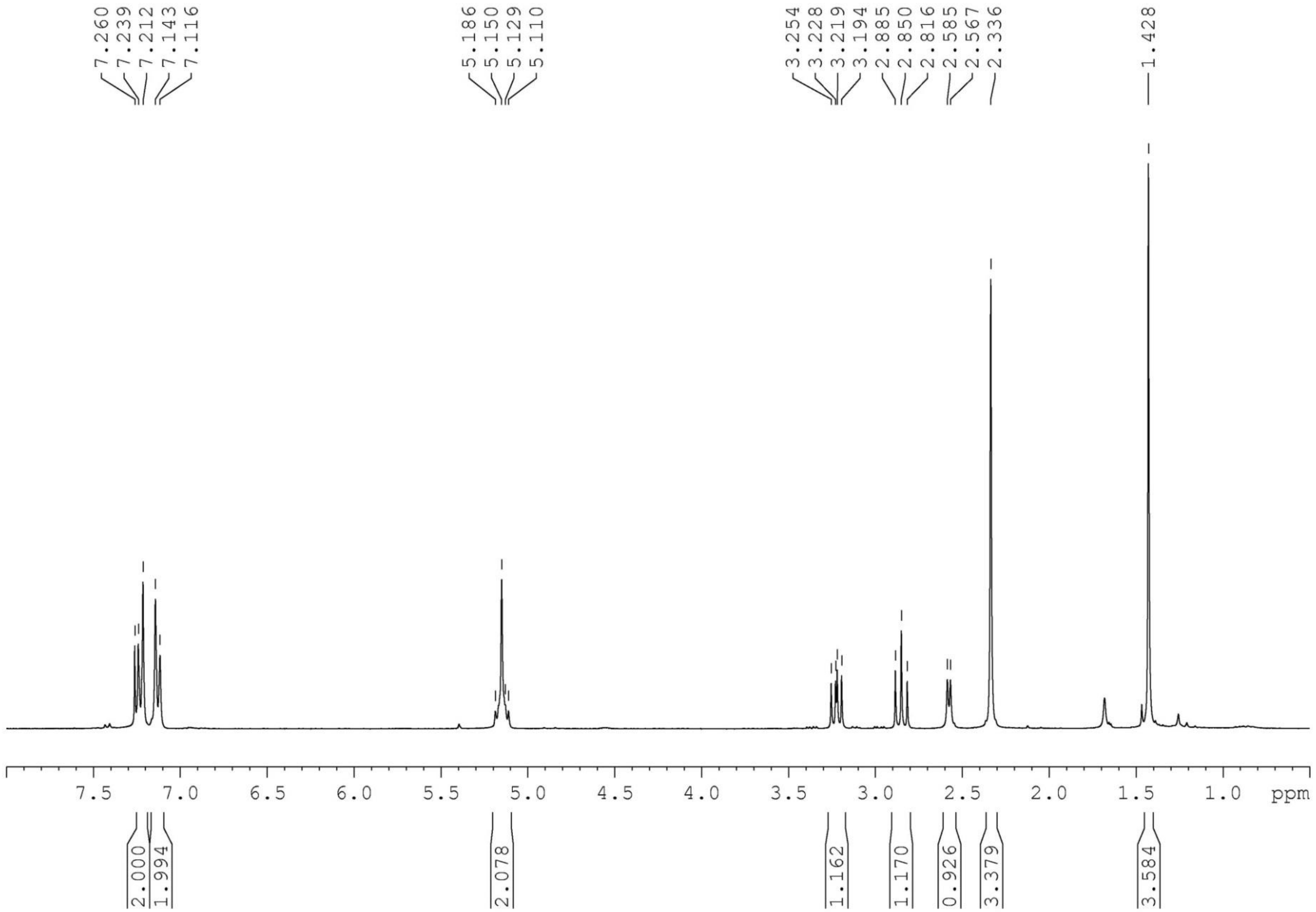

Compound **7c** ^{13}C NMR in $CDCl_3$ (75 MHz)

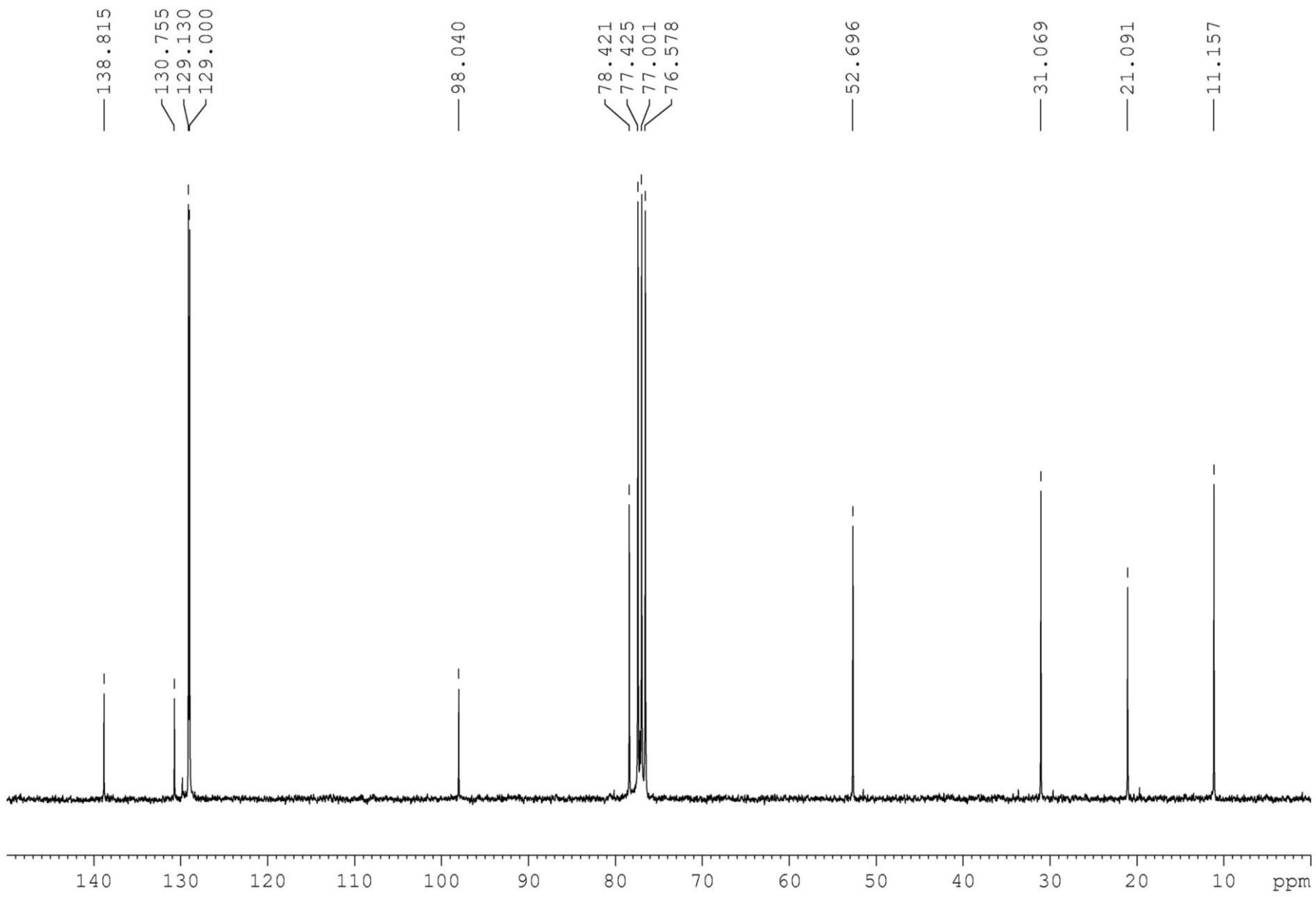

Compound **8c** ^{1}H NMR in $CDCl_3$ (400 MHz)

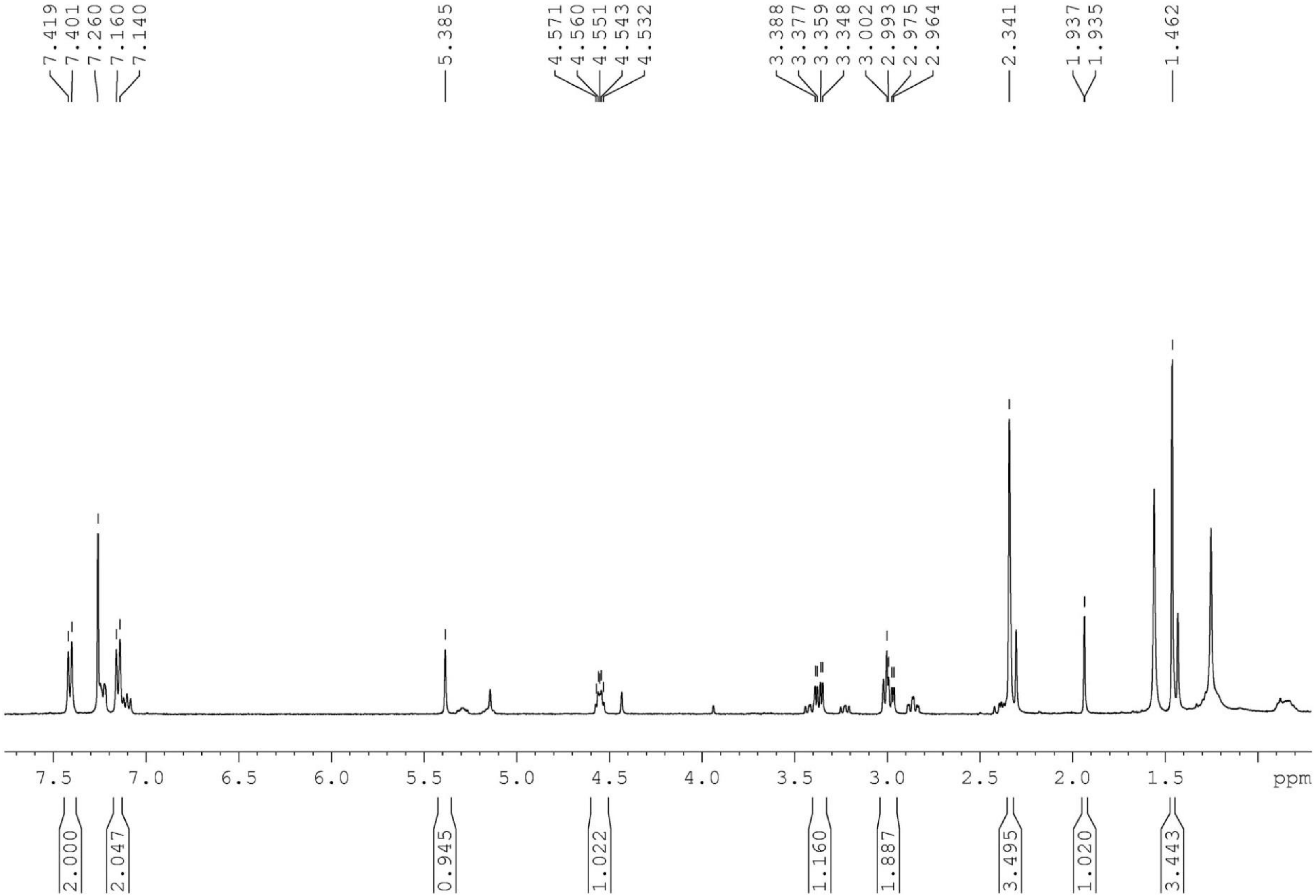

Compound **8c** ^{13}C NMR in $CDCl_3$ (100 MHz)

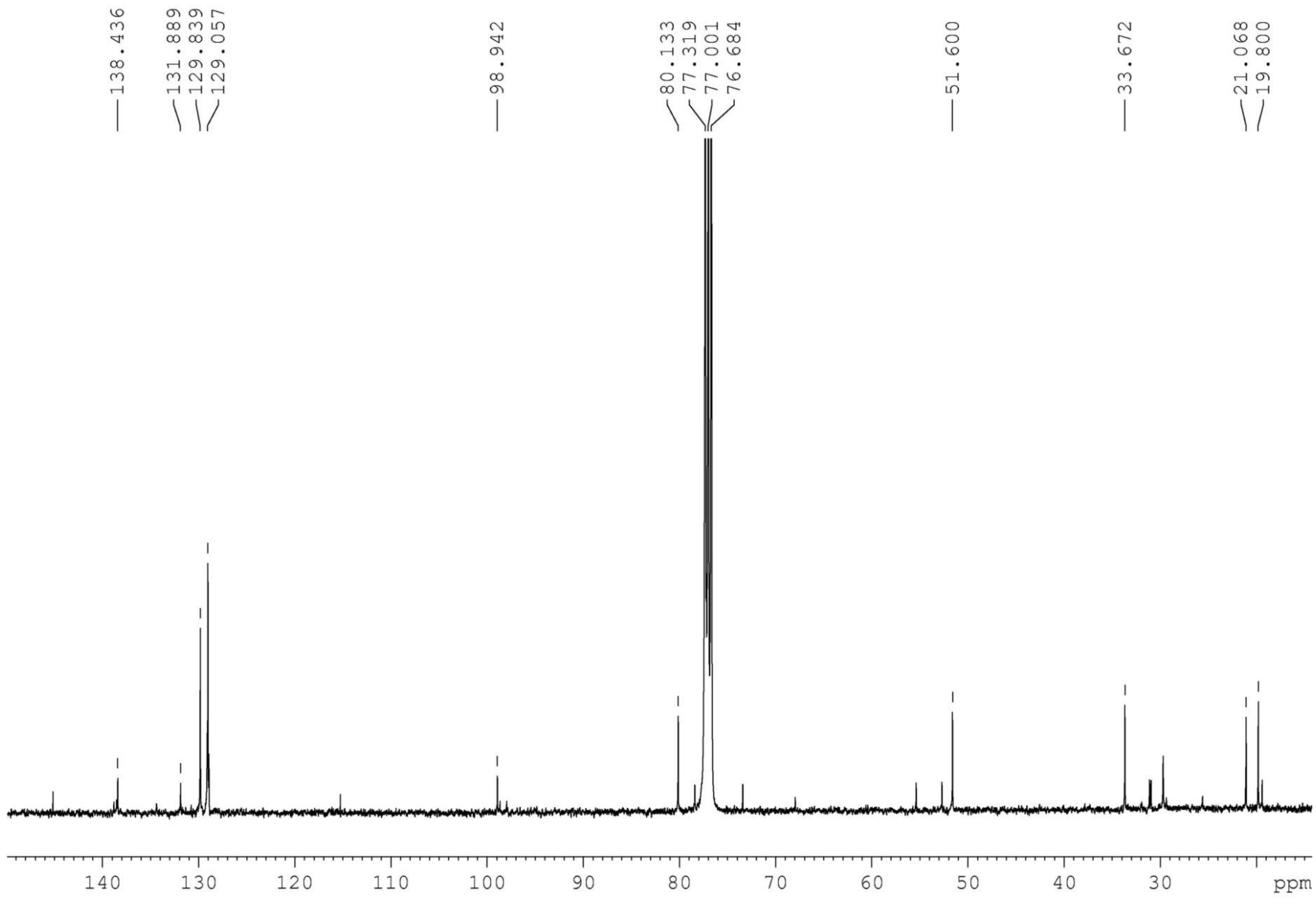

Compound **7d** ^{1}H NMR in $CDCl_3$ (300 MHz)

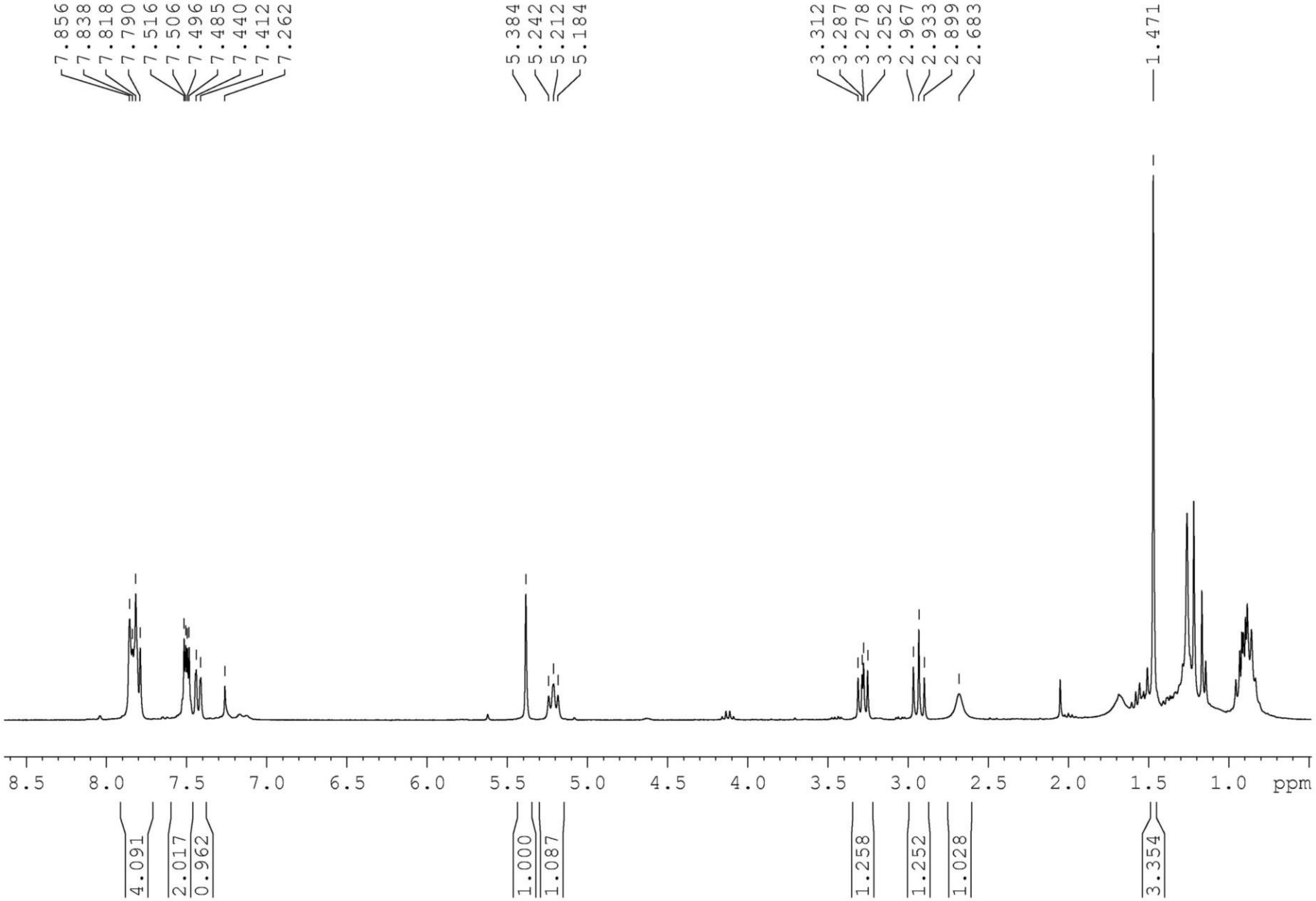

Compound **7d** ^{13}C NMR in $CDCl_3$ (100 MHz)

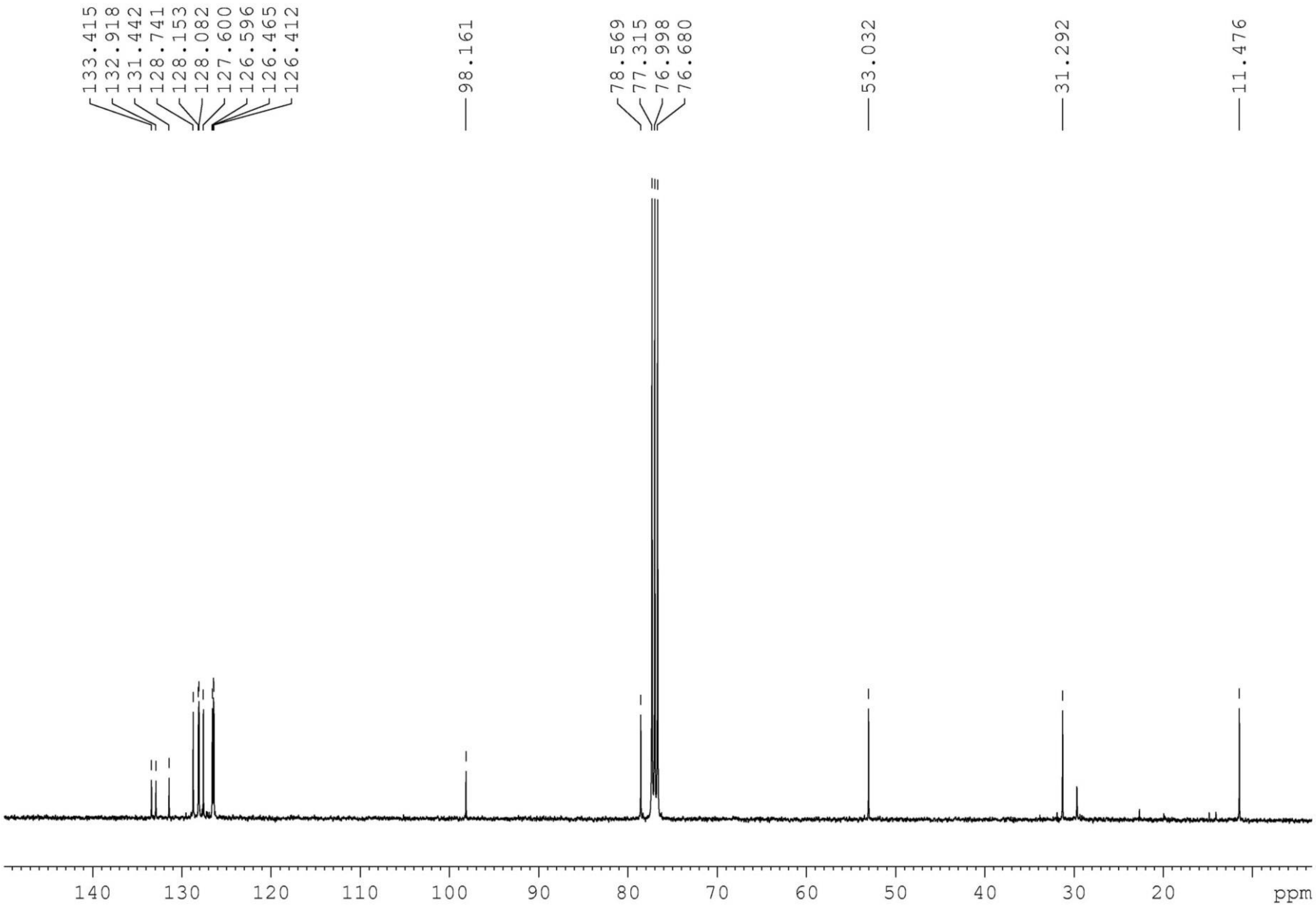

Mixture of compounds **8d** and **9d** ^{1}H NMR in $CDCl_3$ (250 MHz)

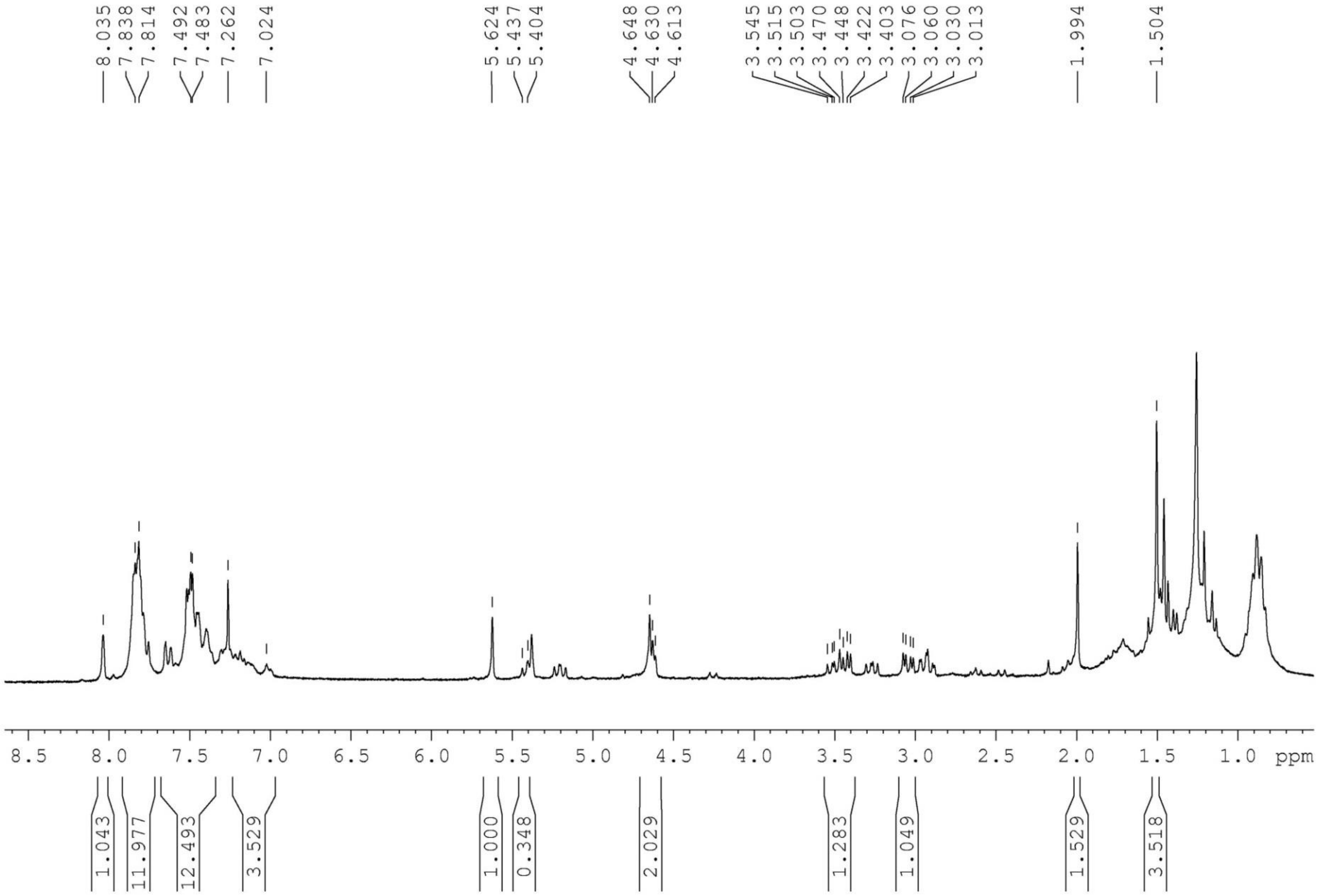

Mixture of compounds **8d** and **9d** ^{13}C NMR in $CDCl_3$ (62.5 MHz)

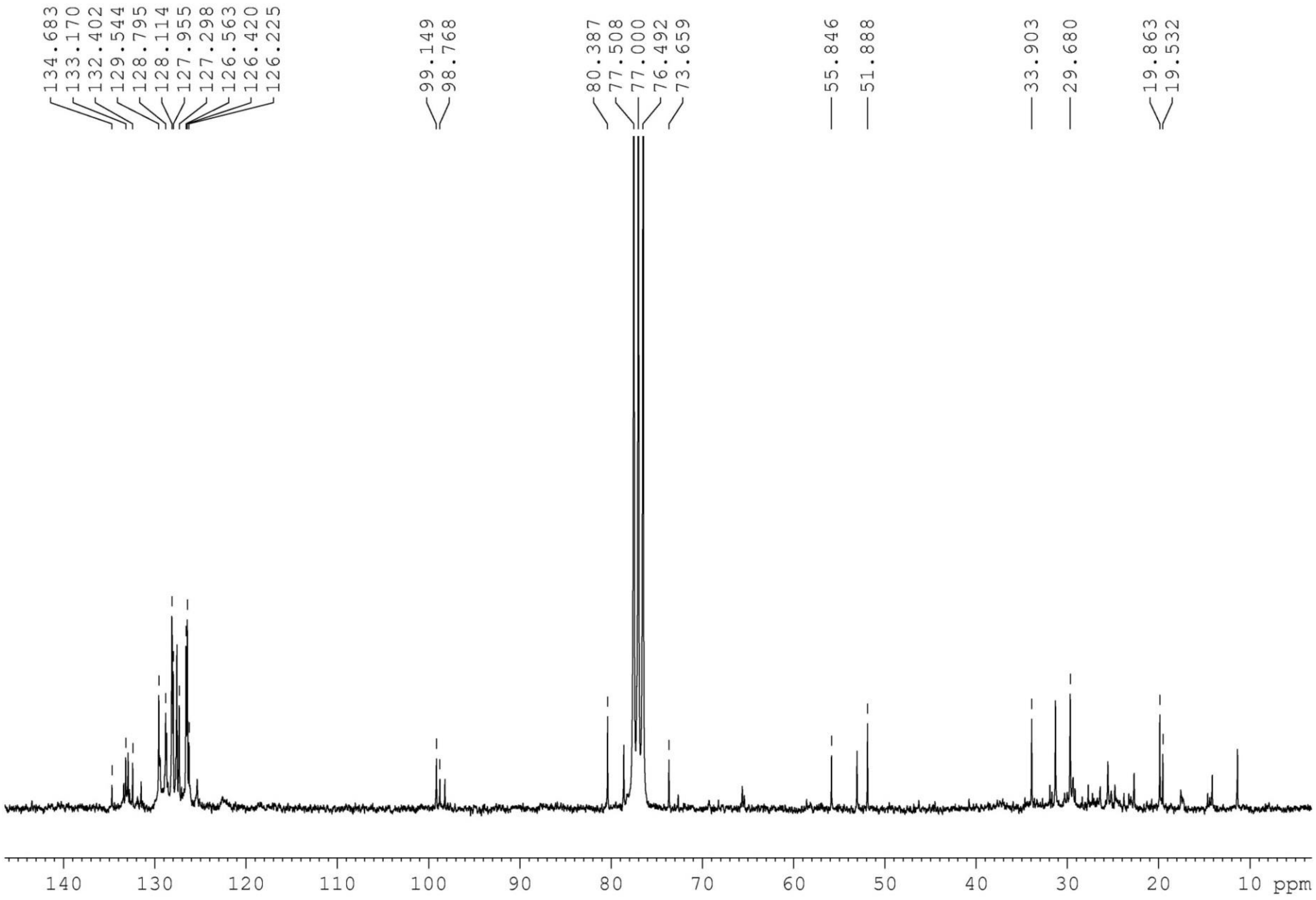

Confirmation of the relative configuration of compounds 7a, 8a and 9a by NOESY and NOE
(600 MHz, $CDCl_3$)

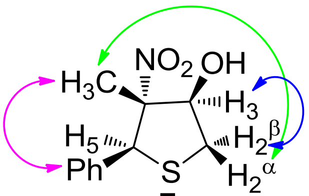

7a

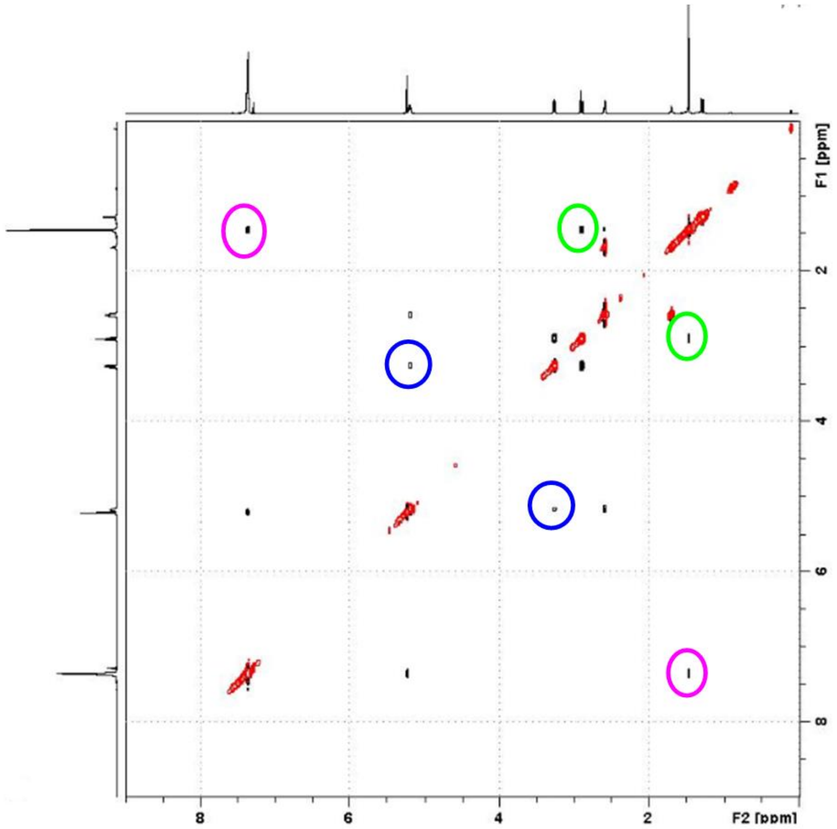

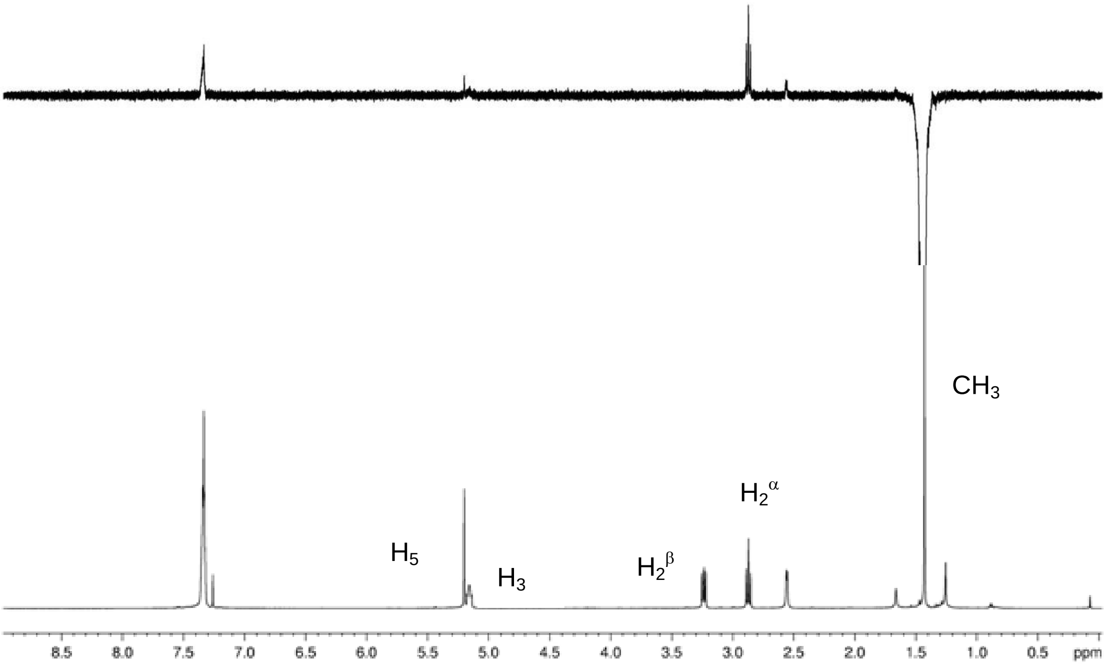

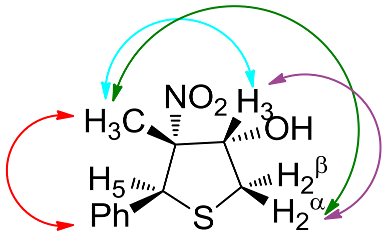
H3C
NO2 H3
OH
H5
Ph
S
H2β
H2α

8a

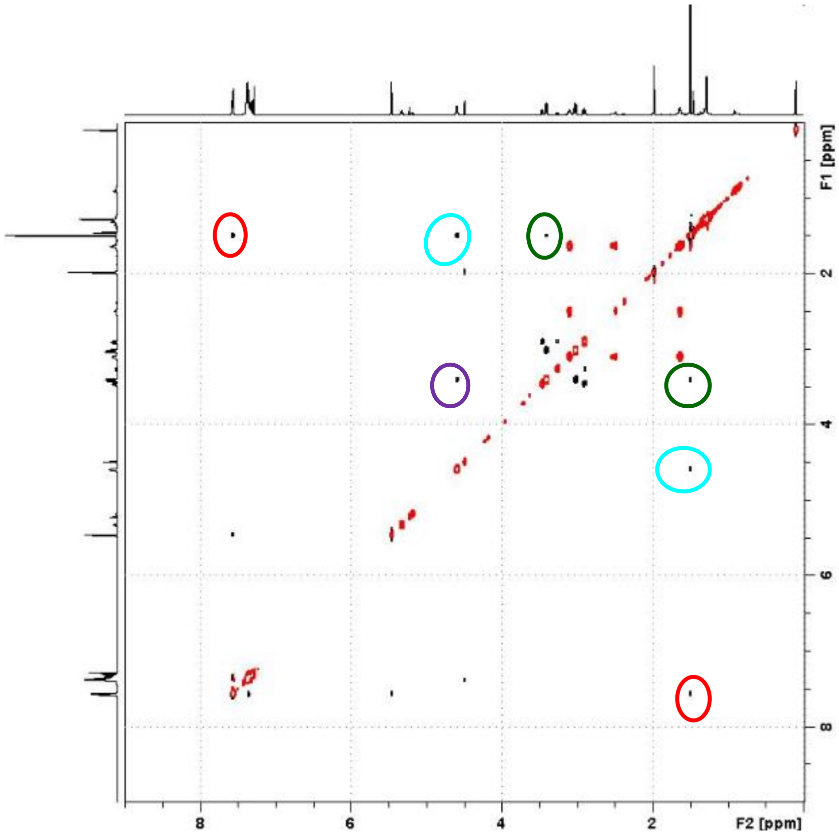
F1 [ppm]
2
4
6
8
F2 [ppm]

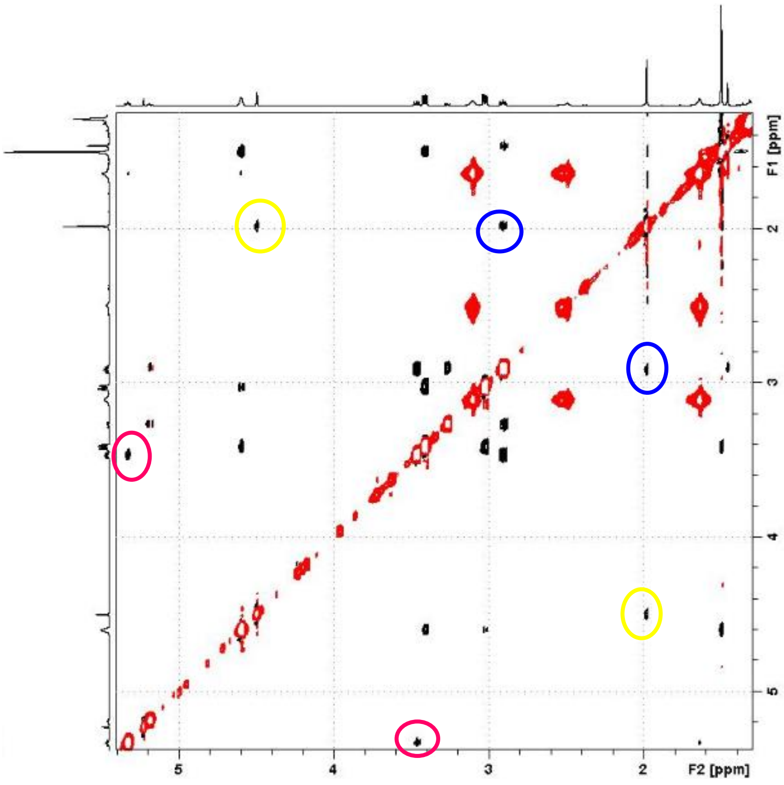
F1 [ppm]
2
3
4
5
4
3
2
F2 [ppm]

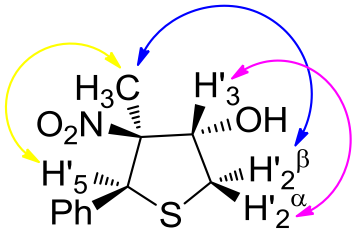
H3C
H'3
O2N
OH
H'5
Ph
S
H'2β
H'2α

9a

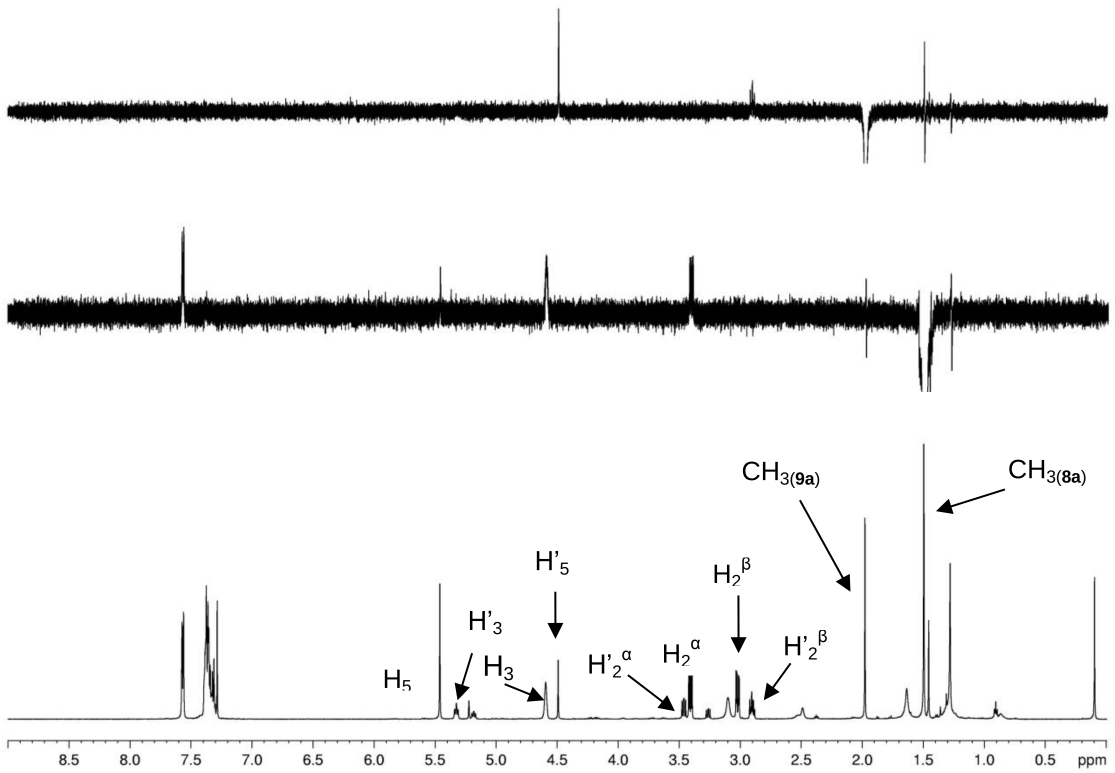
CH3(9a)
CH3(8a)
H'5
H2β
H'3
H'2β
H3
H'2α
H2α
H5
8.5
8.0
7.5
7.0
6.5
6.0
5.5
5.0
4.5
4.0
3.5
3.0
2.5
2.0
1.5
1.0
0.5
ppm

HPLC Chromatograms

Compound 5

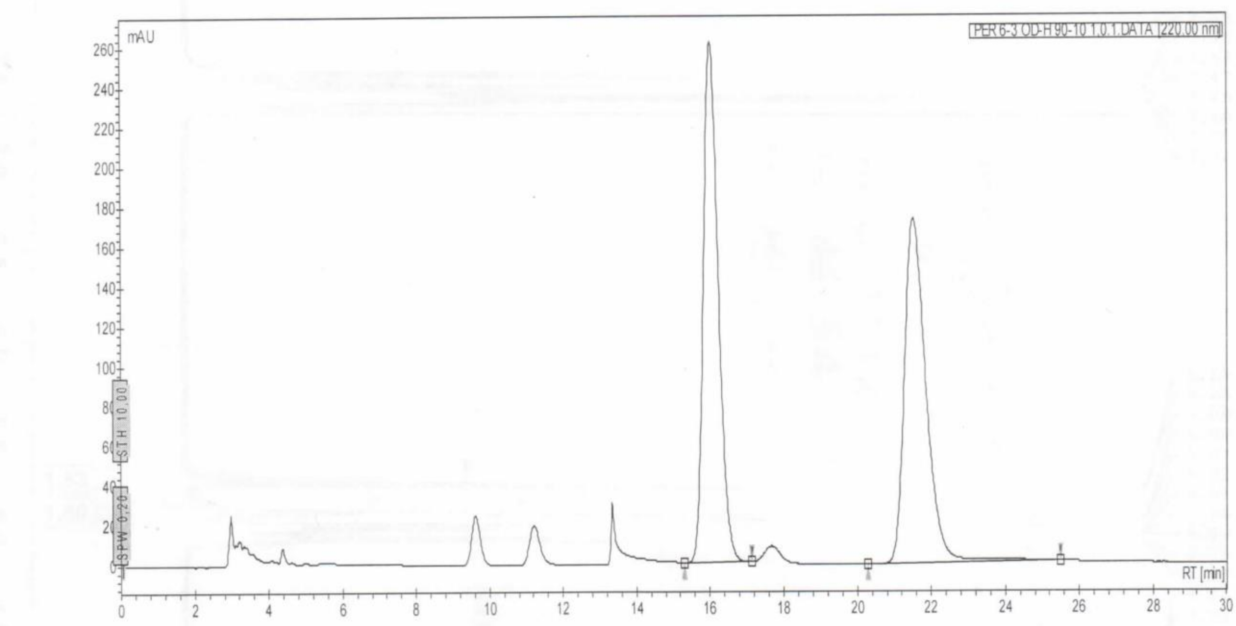

Peak results :

PER 6-3 OD-H 90-10 1,0.1.DATA [220,00 nm]

Index	Name	Time [Min]	Quantity [% Area]	Height [mAU]	Area [mAU.Min]	Area % [%]
1	UNKNOWN	16,012	51,02	260,8	122,4	51,025
2	UNKNOWN	21,518	48,98	172,8	117,5	48,975
Total			100,00	433,6	239,9	100,000

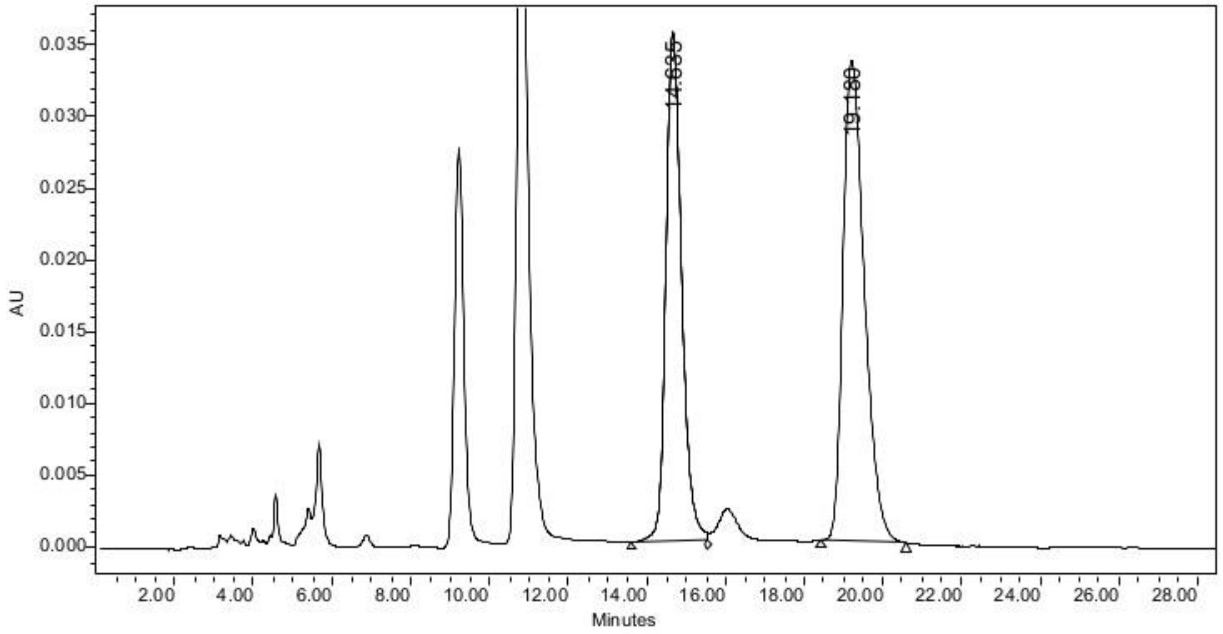

	RT (min)	Area (V*sec)	% Area	Height (V)	% Height
1	14.635	990351	43.86	35411	51.41
2	19.180	1267668	56.14	33475	48.59

Compound 6

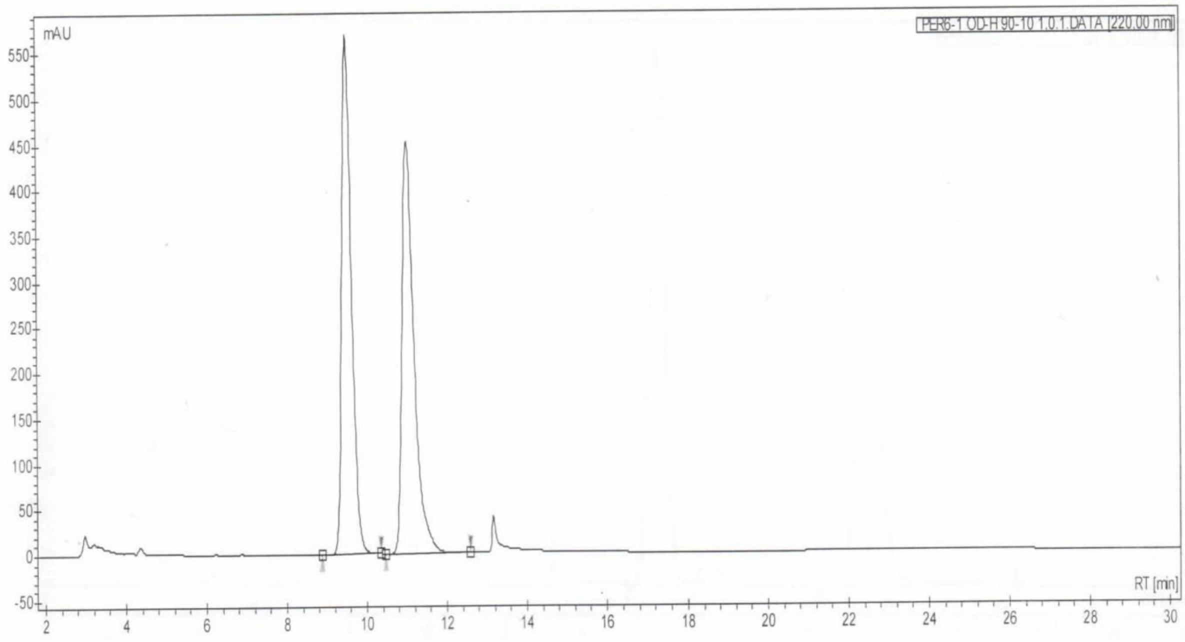

Peak results :

PER6-1 OD-H 90-10 1,0,1.DATA [220,00 nm]

Index	Name	Time [Min]	Quantity [% Area]	Height [mAU]	Area [mAU.Min]	Area % [%]
1	UNKNOWN	9,506	50,36	570,6	159,7	50,360
2	UNKNOWN	11,013	49,64	452,8	157,5	49,640
Total			100,00	1023,4	317,2	100,000

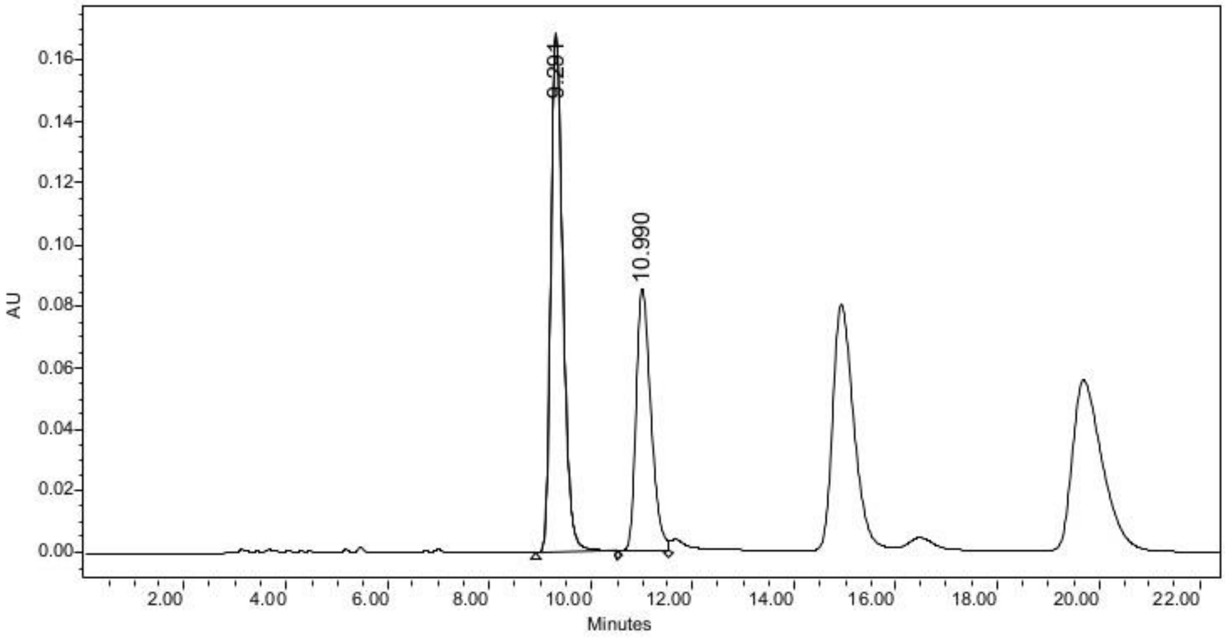

	RT (min)	Area (V*sec)	% Area	Height (V)	% Height
1	9.291	2836518	62.02	169304	66.49
2	10.990	1737071	37.98	85340	33.51

Compound 7a

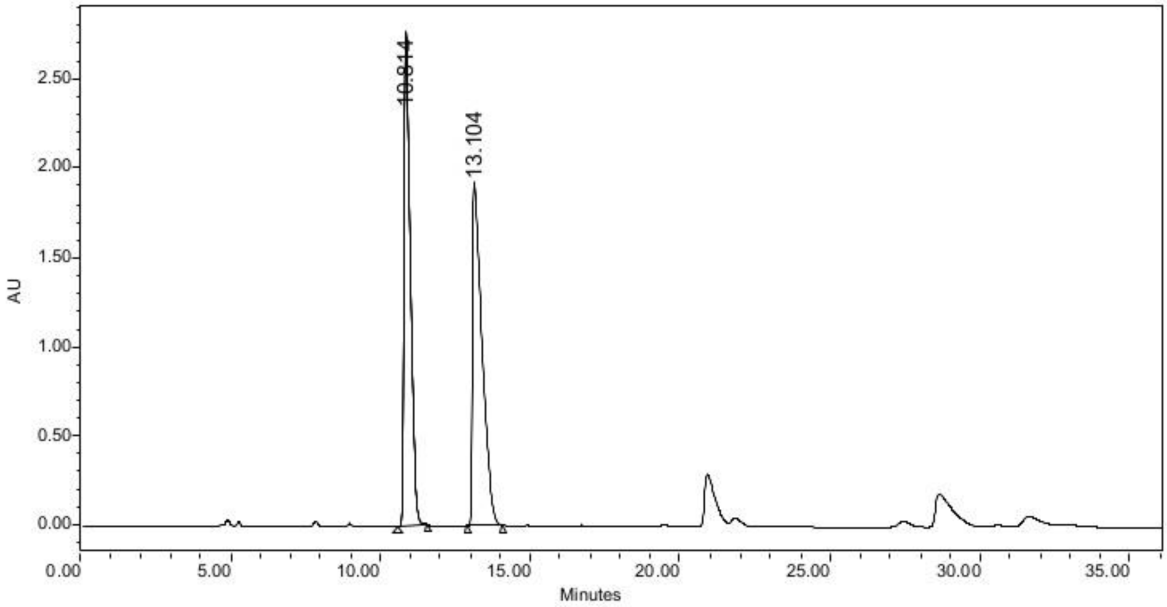

	RT (min)	Area (V*sec)	% Area	Height (V)	% Height
1	10.814	41287261	49.12	2774209	58.99
2	13.104	42768226	50.88	1928687	41.01

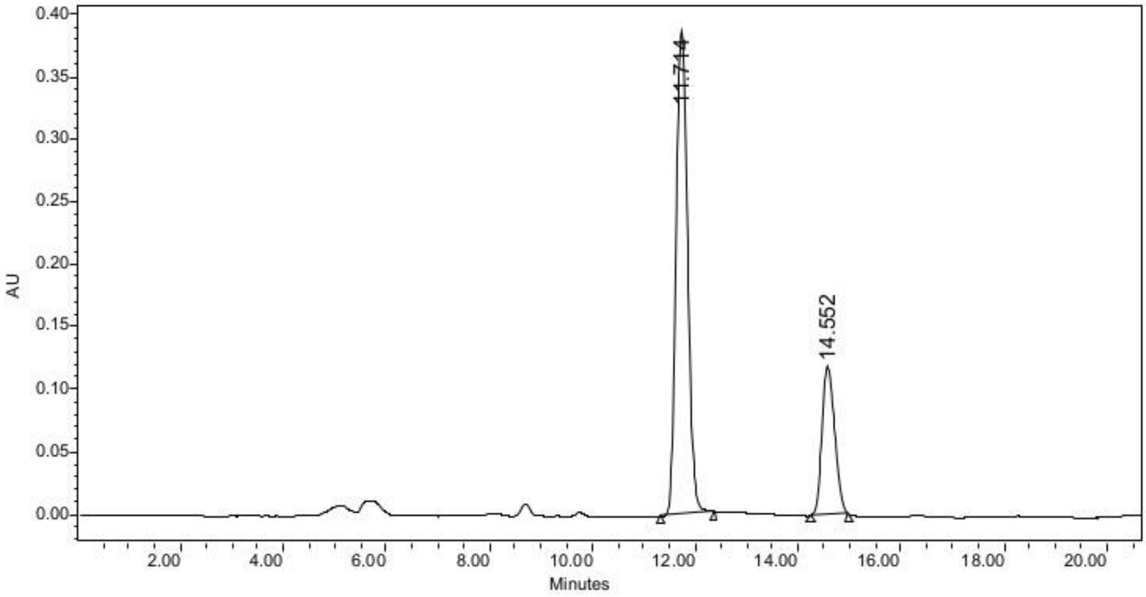

	RT (min)	Area (V*sec)	% Area	Height (V)	% Height
1	11.714	6069269	74.92	386875	76.49
2	14.552	2031640	25.08	118928	23.51

Compound 11

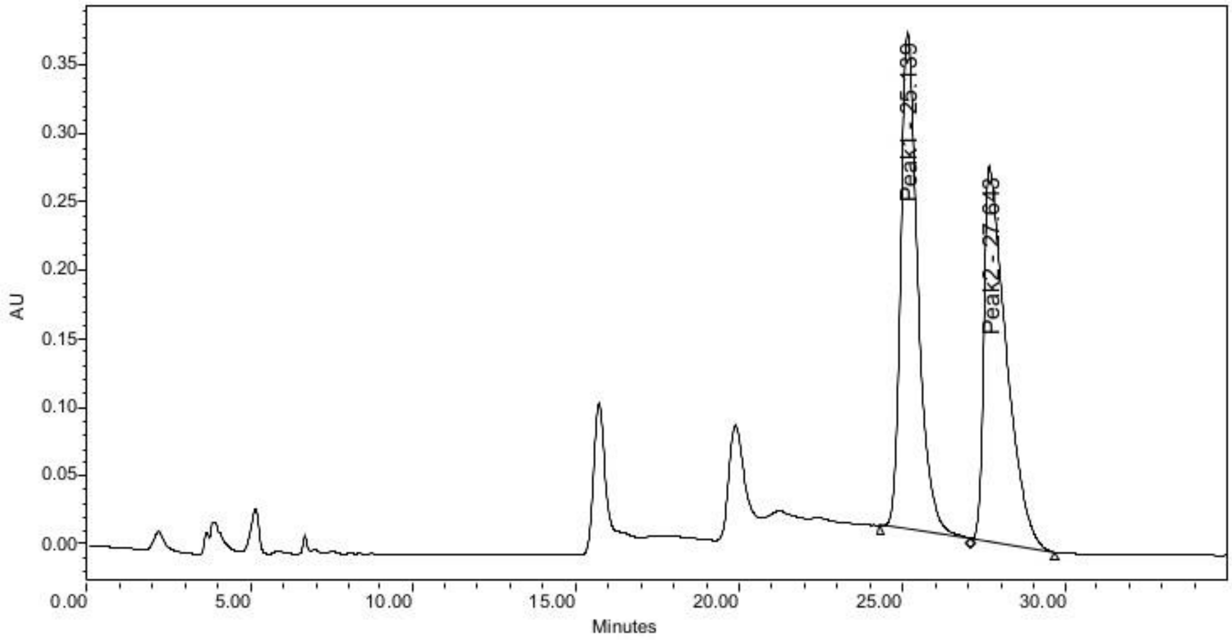

	Peak Name	RT (min)	Area (V*sec)	% Area	Height (V)	% Height
1	Peak1	25.139	13439238	50.61	362983	56.90
2	Peak2	27.643	13114088	49.39	274946	43.10

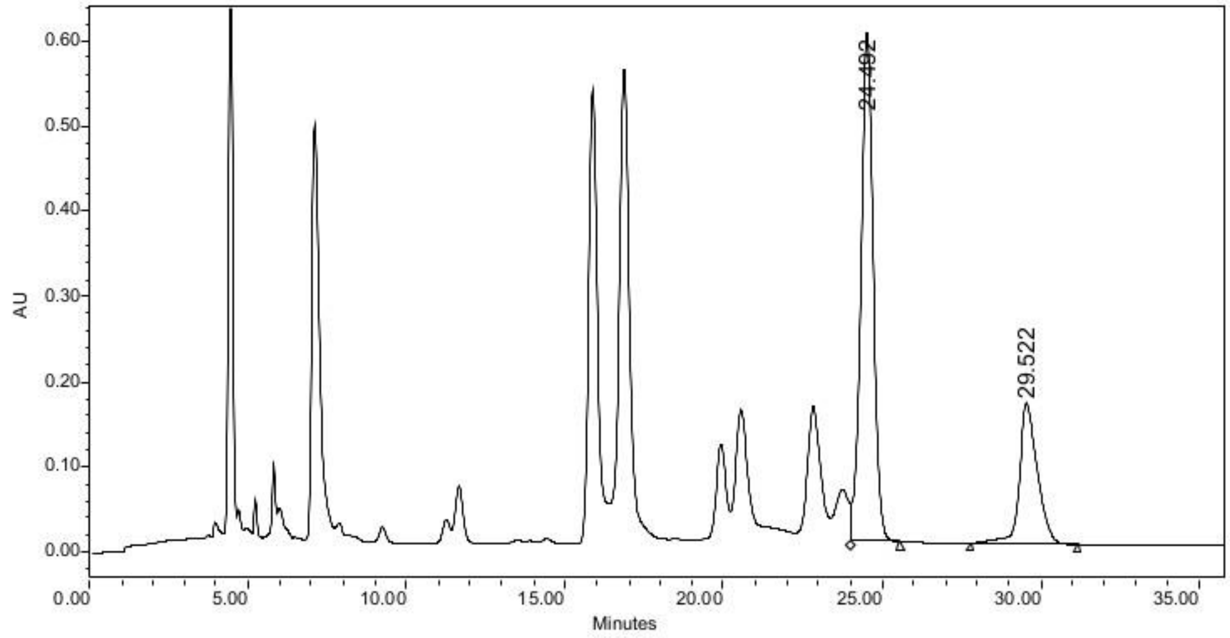

	RT (min)	Area (V*sec)	% Area	Height (V)	% Height
1	24.492	16210928	71.46	597882	78.36
2	29.522	6473528	28.54	165158	21.64

Compound 7b

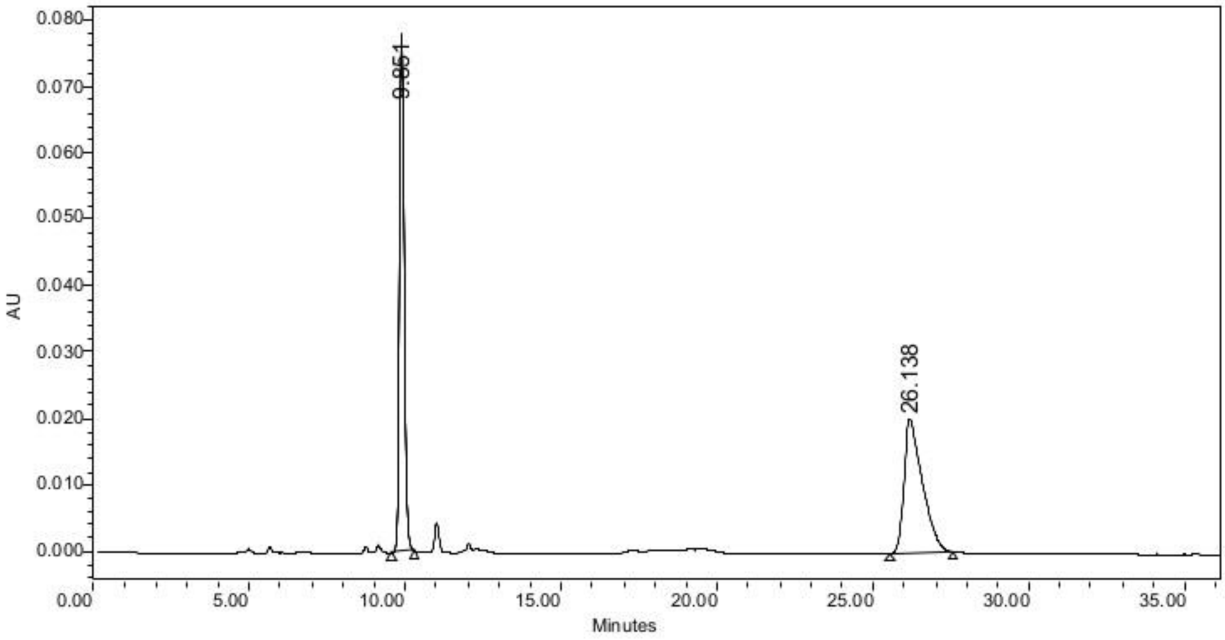

	RT (min)	Area (V*sec)	% Area	Height (V)	% Height
1	9.851	834109	50.23	78119	79.39
2	26.138	826474	49.77	20286	20.61

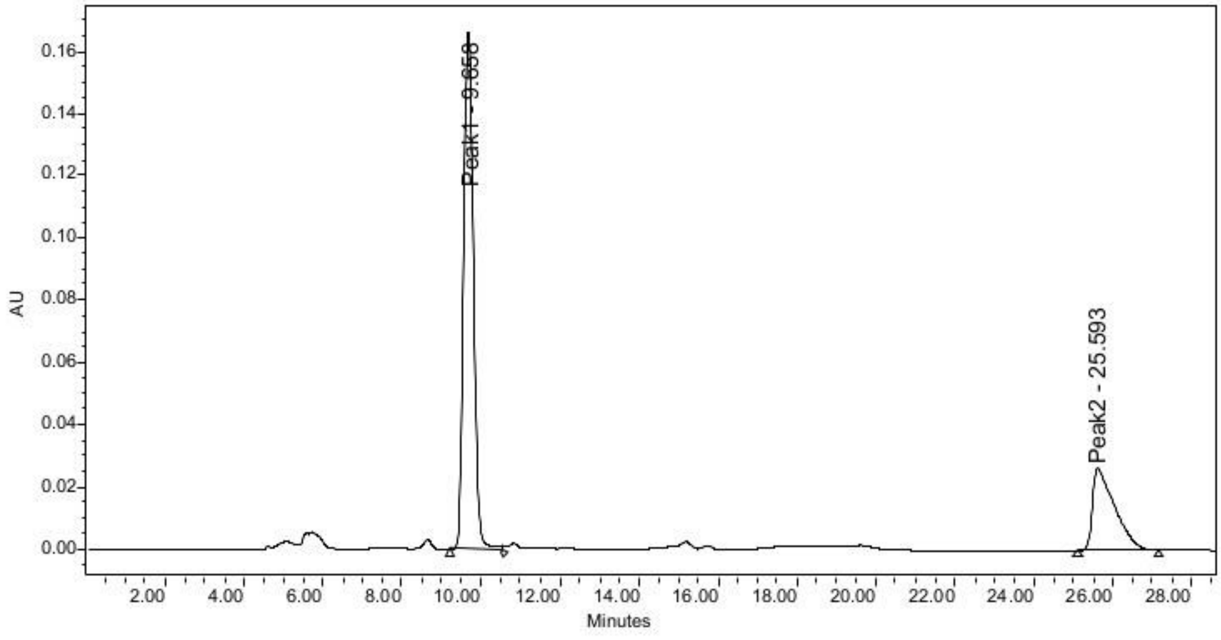

	Peak Name	RT (min)	Area (V*sec)	% Area	Height (V)	% Height
1	Peak1	9.658	2904249	73.82	166409	86.30
2	Peak2	25.593	1029766	26.18	26420	13.70

Compound 7c

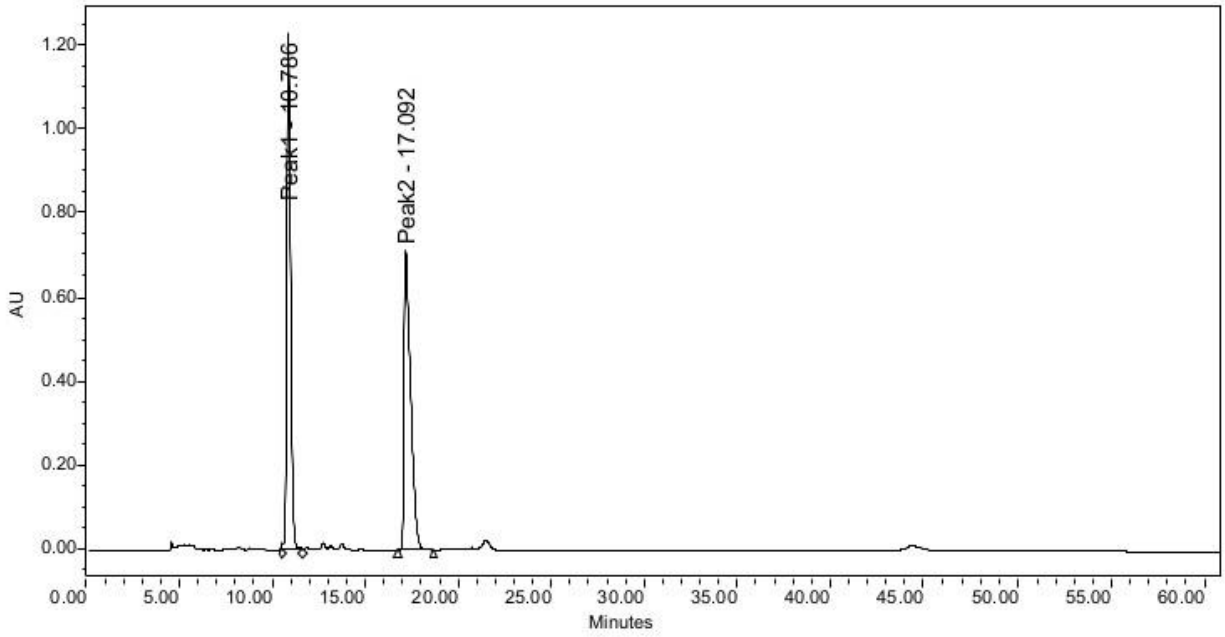

	Peak Name	RT (min)	Area (V*sec)	% Area	Height (V)	% Height
1	Peak1	10.786	17910851	50.45	1231535	63.24
2	Peak2	17.092	17590409	49.55	715932	36.76

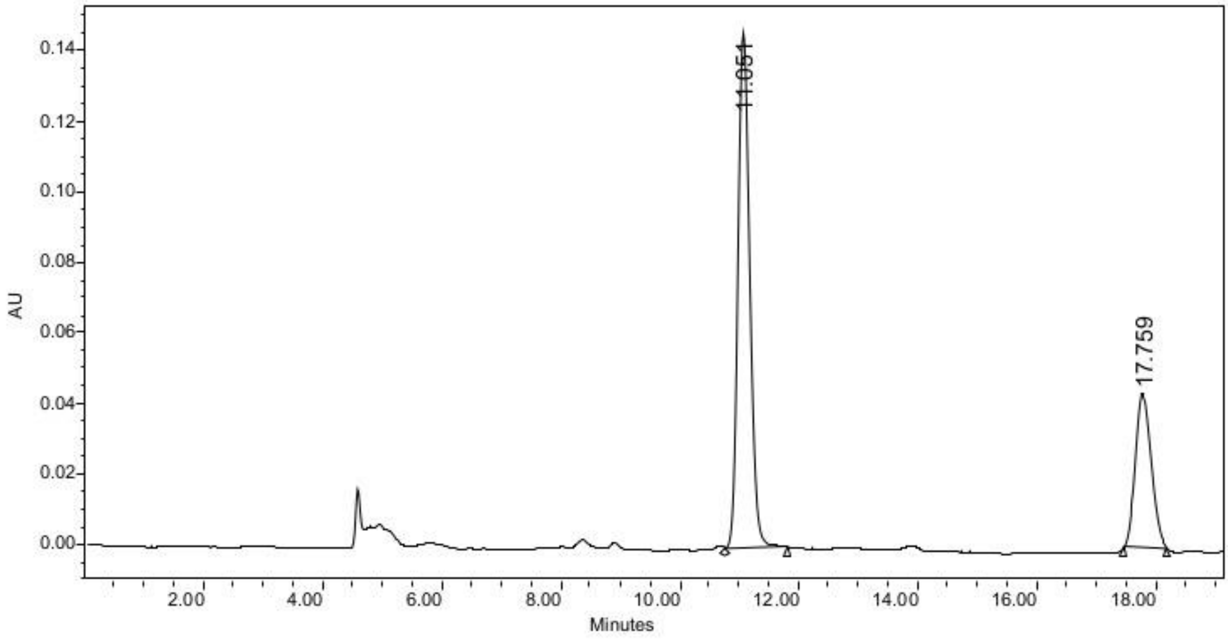

	RT (min)	Area (V*sec)	% Area	Height (V)	% Height
1	11.051	2025382	71.21	146301	76.91
2	17.759	818755	28.79	43916	23.09

Compound 7d

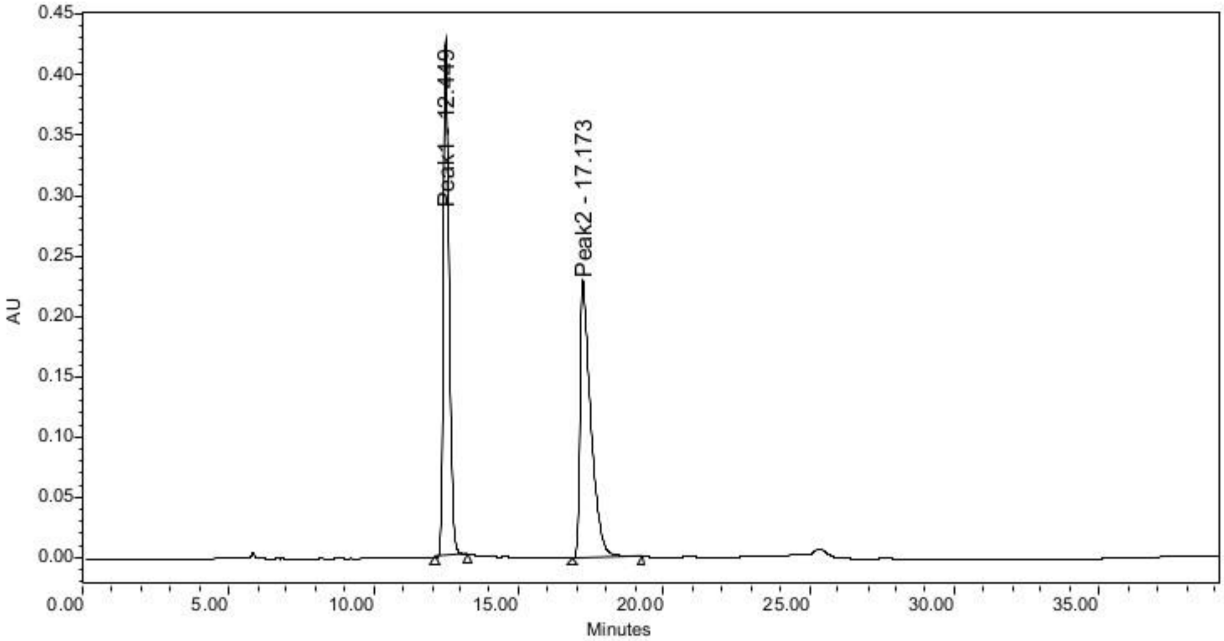

	Peak Name	RT (min)	Area (V*sec)	% Area	Height (V)	% Height
1	Peak1	12.449	5955265	49.96	428098	65.07
2	Peak2	17.173	5964433	50.04	229779	34.93

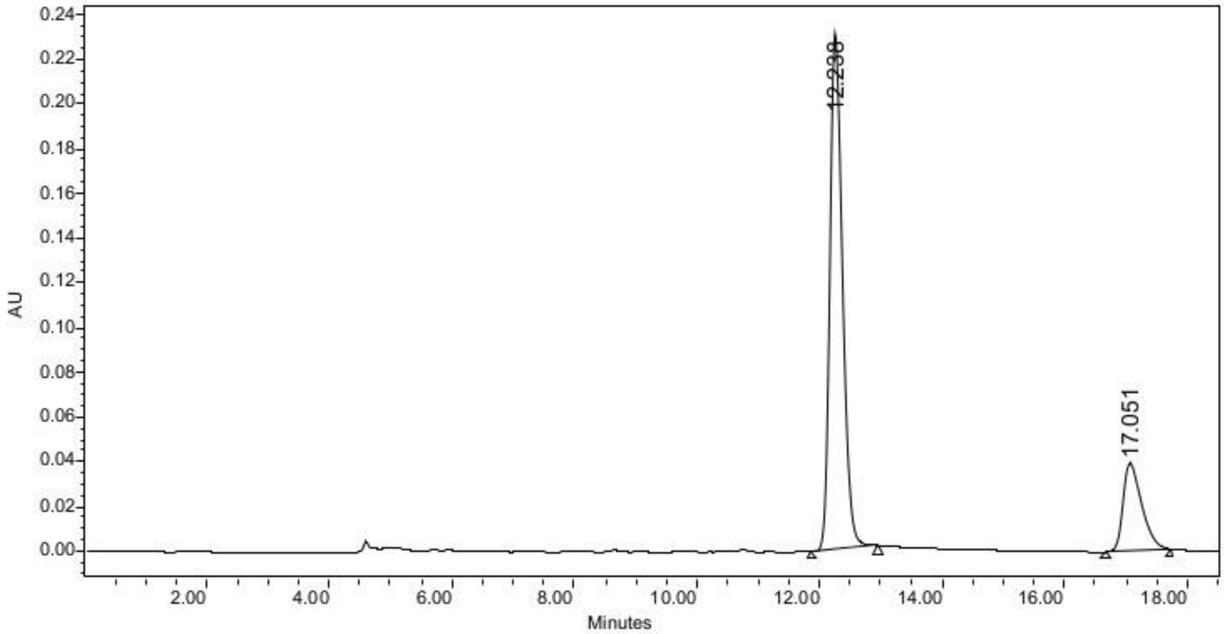

	RT (min)	Area (V*sec)	% Area	Height (V)	% Height
1	12.238	3298969	79.82	231405	85.49
2	17.051	834193	20.18	39290	14.51
